# Diagnosis, prevalence, and clinical impact of sarcopenia in COPD: a systematic review and meta‐analysis

**DOI:** 10.1002/jcsm.12600

**Published:** 2020-08-30

**Authors:** Walter Sepúlveda‐Loyola, Christian Osadnik, Steven Phu, Andrea A. Morita, Gustavo Duque, Vanessa S. Probst

**Affiliations:** ^1^ Department of Physiotherapy Londrina State University Londrina Paraná Brazil; ^2^ Centre of Research and Post‐Graduation in Health Sciences (CEPPOS) Londrina State University Londrina Paraná Brazil; ^3^ Department of Physiotherapy Monash University Melbourne VIC Australia; ^4^ Monash Lung and Sleep, Monash Health Monash Medical Centre Melbourne VIC Australia; ^5^ Department of Medicine—Western Health, Melbourne Medical School The University of Melbourne St Albans VIC Australia; ^6^ Australian Institute for Musculoskeletal Science (AIMSS) University of Melbourne and Western Health St Albans VIC Australia

**Keywords:** Sarcopenia, COPD, Prevalence, Diagnosis, Aging

## Abstract

Sarcopenia prevalence and its clinical impact are reportedly variable in chronic obstructive pulmonary disease (COPD) due partly to definition criteria. This review aimed to identify the criteria used to diagnose sarcopenia and the prevalence and impact of sarcopenia on health outcomes in people with COPD. This review was registered in PROSPERO (CRD42018092576). Five electronic databases were searched to August 2018 to identify studies related to sarcopenia and COPD. Study quality was assessed using validated instruments matched to study designs. Sarcopenia prevalence was determined using authors' definitions. Comparisons were made between people who did and did not have sarcopenia for pulmonary function, exercise capacity, quality of life, muscle strength, gait speed, physical activity levels, inflammation/oxidative stress, and mortality. Twenty‐three studies (70% cross‐sectional) from Europe (10), Asia (9), and North and South America (4) involving 9637 participants aged ≥40 years were included (69.5% men). Sarcopenia criteria were typically concordant with recommendations of hEuropean and Asian consensus bodies. Overall sarcopenia prevalence varied from 15.5% [95% confidence interval (CI) 11.8–19.1; combined muscle mass, strength, and/or physical performance criteria] to 34% (95%CI 20.6–47.3; muscle mass criteria alone) (*P* = 0.009 between subgroups) and was greater in people with more severe [37.6% (95%CI 24.8–50.4)] versus less severe [19.1% (95%CI 10.2–28.0)] lung disease (*P* = 0.020), but similar between men [41.0% (95%CI 26.2–55.9%)] and women [31.9% (95%CI 7.0–56.8%)] (*P* = 0.538). People with sarcopenia had lower predicted forced expiratory volume in the first second (mean difference −7.1%; 95%CI −9.0 to −5.1%) and poorer exercise tolerance (standardized mean difference −0.8; 95%CI −1.4 to −0.2) and quality of life (standardized mean difference 0.26; 95%CI 0.2–0.4) compared with those who did not (*P* < 0.001 for all). No clear relationship was observed between sarcopenia and inflammatory or oxidative stress biomarkers. Incident mortality was unreported in the literature. Sarcopenia is prevalent in a significant proportion of people with COPD and negatively impacts upon important clinical outcomes. Opportunities exist to optimize its early detection and management and to evaluate its impact on mortality in this patient group.

## Introduction

Chronic obstructive pulmonary disease (COPD) is a condition characterized by chronic inflammation^1^ and extrapulmonary changes that negatively affect physical function (e.g. lower levels of physical activity^2^ and reductions in muscle mass and strength^3^, ^4^) and quality of life.^5^, ^6^ The presence of such factors is also closely related to the presence of sarcopenia,^7^ a syndrome characterized by lower muscle mass, muscle strength, and physical performance.^7^ Sarcopenia is a significant contributor to frailty in the elderly population and is associated with increased rates of falls, hospitalization, and mortality.^8^, ^9^ It has been estimated to occur in approximately 5–13% of the ‘healthy’ older population.^4^, ^7^

People with COPD appear to have an increased risk of developing sarcopenia, with prevalence estimates ranging from 15%^2^ to 55%.^10^ In this patient group, sarcopenia appears to confer a negative impact upon clinical outcomes related to function and health^1^, ^3^, ^11^, ^12^, ^13^ and its prevalence appears to increase with increasing COPD‐related impairment. Although sarcopenia has also been shown to contribute towards poorer prognosis in people with COPD,^2^, ^3^ the real clinical impact has not yet been analysed. Additionally, the wide‐ranging prevalence estimates of sarcopenia in COPD, however, make its true impact somewhat difficult to accurately ascertain.

A significant factor contributing to this large variability appears to be choice of definition criteria.^2^, ^3^, ^14^ International recommendations exist for the diagnosis of sarcopenia in older people such as those proposed by the European Working Group of Sarcopenia in Older People (EWGSOP)^7^ and the Asian Group of Sarcopenia,^15^ yet these have not been featured in published literature in the field of COPD. Considering the prevalence of both sarcopenia and COPD increase with increasing age, the impact of sarcopenia on a broader range of clinically important COPD‐related outcomes is also not currently clear. This review therefore aimed to evaluate the literature pertaining specifically to people with COPD to identify the criteria used to diagnose sarcopenia, estimate its prevalence, and evaluate its impact upon health outcomes.

## Methodology

### Data sources and search strategy

The protocol for this review was registered in PROSPERO (CRD42018092576). Five electronic databases (i.e. PubMed, LILACS, EMBASE, The Cochrane Library, and Scielo) were searched from inception until August 2018 using the following free‐text and subject heading terms: ‘COPD’, ‘pulmonary disease, chronic obstructive’, ‘chronic obstructive lung disease’, ‘COAD’, ‘chronic obstructive airway disease’, and ‘sarcopeni*’ (Supporting Information, *Table* S1). Hand searching of reference lists from included articles was also conducted to identify additional potential studies. To be eligible for inclusion, studies must have been conducted on adults with COPD (aged ≥40 years), defined according to authors, irrespective of disease severity (GOLD: Global Strategy for the Diagnosis, Management and Prevention of COPD, Global Initiative for Chronic Obstructive Lung Disease^16^ and reported upon a diagnosis of sarcopenia, defined according to any criteria provided it was stated in the methodology. Considering the nature of our research question, we included observational (e.g. cohort) and cross‐sectional studies and clinical trials (whether randomized or not). Abstracts and publications published in languages other than English, Spanish, or Portuguese were not eligible for inclusion.

The principal outcomes for this review were (i) the criteria used to define sarcopenia and its prevalence and (ii) clinical data from studies that provided comparative data between people with COPD who did and did not have sarcopenia, as follows: (a) quality of life, from either generic or respiratory‐specific quality of life questionnaires; (b) physical function, derived from common clinical tests of exercise capacity, muscle strength, and balance; (c) physical activity levels, measured by objective physical activity monitors; (d) pulmonary function, measured by spirometry (e.g. FEV_1_% predicted); (e) inflammatory or oxidative stress biomarkers [e.g. interleukin (IL)‐6, tumour necrosis factor‐alpha, C‐reactive protein, catalase, paraxonase‐1]; and (f) all‐cause mortality.

### Data management and quality appraisal

Database search yields were collated within a bibliographical reference manager software (StArt v.3.03^17^), and duplicates were discarded. Citations were screened for eligibility upon title and abstract by two independent reviewers (W.S.L and A.A.M) and classified as either ‘include’, ‘exclude’, or ‘maybe’. Those deemed ‘include’ or ‘maybe’ were reviewed in full text to derive a final yield, with any disagreements resolved via a third, independent assessor (V.S.P). This process was summarized in accordance with Preferred Reporting Items for Systematic Reviews and Meta‐Analyses recommendations.^18^ Data were extracted by two members of the team (W.S.L and A.A.M) using standardized templates appropriate for the study objectives.

Study quality was appraised using validated instruments tailored according to study design, as follows: (i) National Institutes of Health Quality Assessment Tool for Observational Cohort and Cross‐Sectional Studies, to assess the quality of cohort and cross‐sectional studies; (ii) PEDro scale to assess the quality of randomized clinical trials; and (iii) Joanna Briggs Institute Critical Appraisal Checklist for Quasi‐Experimental Studies to assess the quality of non‐randomized controlled trials.

### Statistical analysis

An overall estimate of sarcopenia prevalence was derived by pooling the proportion of patients with COPD who had detected sarcopenia in individual studies in a meta‐analysis. For this purpose, only one prevalence estimate was used from each study. Where individual studies reported different types of sarcopenia (e.g. sarcopenia with normal body mass index, sarcopenic obesity, severe sarcopenia), an aggregated value, if able to be determined, or the most ‘conventional’ type was used. In order to avoid double counting, estimates from individual studies that evaluated sarcopenia via multiple diagnostic criteria (e.g. comparisons of different cut‐off thresholds within a single cohort) were pooled using their primary stated method or that which most closely resembled the current EWGSOP recommendation.^7^, ^19^ Where able to be conducted, separate subgroup analyses were conducted to compare prevalence effect estimates between sarcopenia definitions (1 vs >1 diagnostic criteria), gender (male versus female), and disease severity (GOLD I–II versus III–IV), evaluated via χ^2^ test. This meta‐analysis was performed via the ‘metaprop’ command in Stata SE 14.2 (Texas, USA) with 95% confidence intervals (CIs) calculated using the score (Wilson) method and a random‐effects model (DerSimonian and Laird method) utilized due to the variability in sarcopenia definitions across studies.

Clinical outcome data from studies comparing people with COPD who did and did not have sarcopenia were meta‐analysed via Review Manager 5.3 (The Nordic Cochrane Centre, The Cochrane Collaboration, Copenhagen 2014). Continuous outcome data evaluated using homogenous metrics (e.g. same test instrument) were summarized as mean differences, while data arising from heterogenous metrics (e.g. same construct, different instrument) were summarized as standardized mean differences (SMDs) and 95%CI. A random‐effects model was used as the principal method of analysis, with statistical heterogeneity described via the *I* ^2^ statistic and interpreted according to Deeks and colleagues (values <25% considered low, 50–75% moderate, and >75% high).^20^

## Results

**Figure 1:**
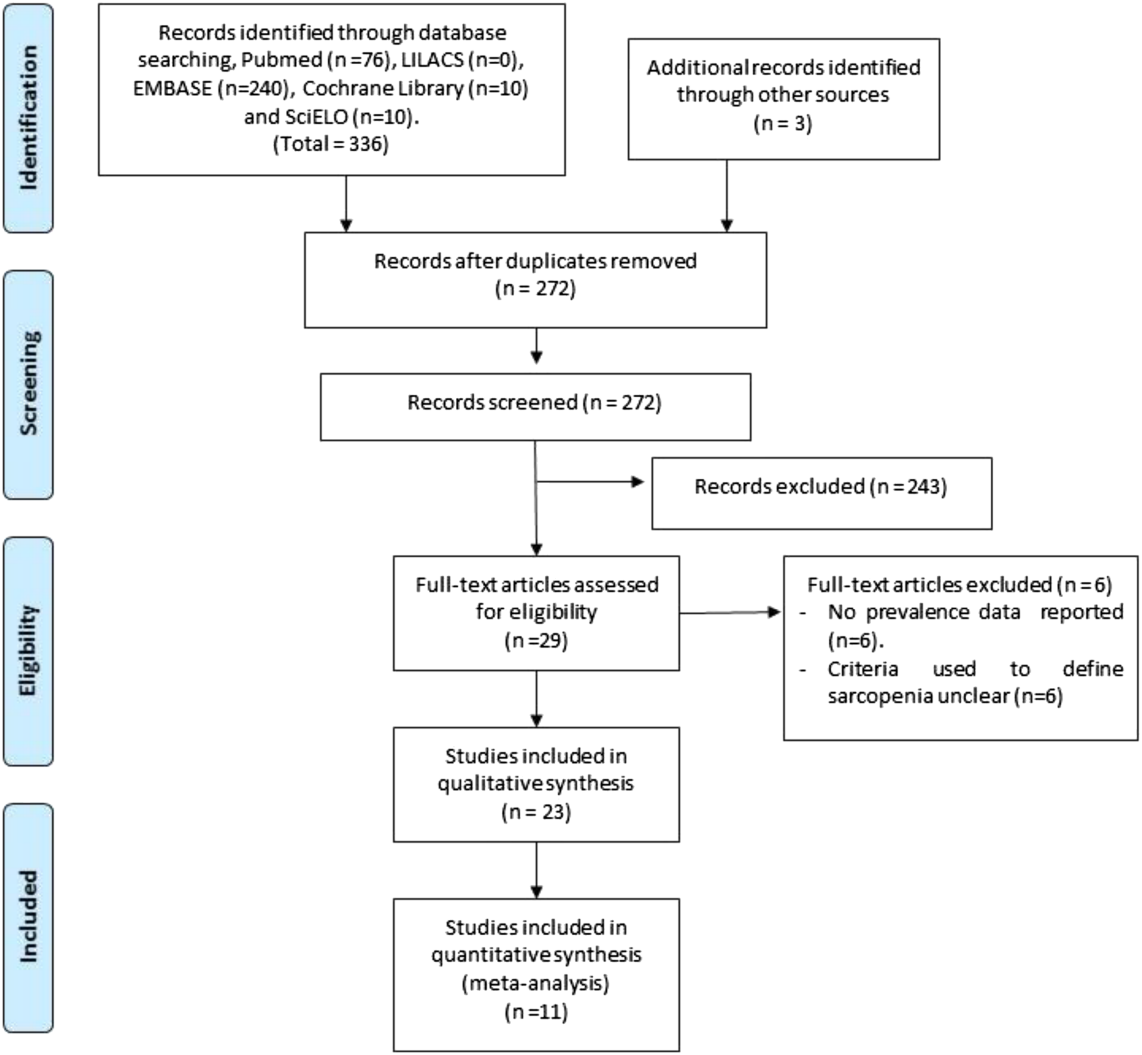
Preferred Reporting Items for Systematic Reviews and Meta‐Analyses flow diagram of article selection.

A detailed summary of the literature search is provided in *Figure* 1. Two hundred and seventy‐two unique records were identified through database searching, resulting in 23 articles involving 9637 participants included in the final review. Of these, seventeen adopted a cross‐sectional design, five were observational cohort studies, and one was a non‐randomized clinical trial. Most studies included patients with differing histories of smoking (those who never smoked and former and current smokers). Comparative data between people with COPD who did and did not have sarcopenia were available from 17 studies. The overall quality of included studies was ‘moderate’ (full details in *Table* S2). Characteristics of included studies are presented in *Table* 1. The review sample spanned a diverse range of populations, including ten studies from Europe, nine from Asia, and four from South America. Most participants were men (69.5%).

**Table 1:** Characteristics of the included studies regarding the prevalence of sarcopenia in subjects with chronic obstructive pulmonary disease

First author and year	Country	Study design	Sample size	Age (mean ± SD)	Male, *n* (%)	Smoking status (never/former/current), *n*	GOLD (%)	Prevalence of sarcopenia		Criteria (assessment method to detect sarcopenia)
								Total, *n* (%)	Male, *n* (%)	
Sergi *et al*. 2006^21^	Italy	Cross‐sectional	40	75.7 ± 5.3	40 (100%)	—	—	15 (38%)	15 (100%)	LMM (DXA)
Koo *et al*. 2014^22^	Korea	Cross‐sectional	574	64.0 ± 0.6	574 (100%)	103/231/240	I/II/III–IV (46/49/5)	155 (27%)	155 (100%)	LMM (DXA)
Gologanu *et al*. 2014^23^	Romania	Cross‐sectional	36	65.6 ± 7.5	12 (33%)	—	I/II/III/IV (0/39/42/19)	3 (8%)	—	LMM (BIA)
Jones *et al*. 2015^2^	UK	Clinical non‐randomized	622	—	354 (57%)	7/170/43	—	90 (14%)	57 (63%)	LMM (BIA)
										LMS (HGS)
										LPP (4MGS)
Costa *et al*. 2015^24^	Brazil	Cross‐sectional	91	67.4 ± 8.7	41 (45%)	91 former smokers	I/II/III/IV (17/24/37/22)	36 (40%)	20 (56%)	LMM (DXA)
Van de Bool *et al*. 2015 ^25^	Netherlands	Retrospective	505	64 (median)	288 (57%)	13/360/132	I/II/III/IV (8/41/40/11)	437 (87%)	239 (55%)	LMM (DXA)
Chung *et al*. 2015^26^	Korea	Retrospective	1039	64.5 ± 9.4 (male) 64.5 ± 10.2 (female)	760 (73%)	129/136/771	I/II/III/IV (46/48/5/1)	283 (27%)	249 (88%)	LMM (DXA)
Joppa *et al*. 2016^27^	ECLIPSE (12 countries and USA)	Cross‐sectional	2000	63.5 ± 7.1	1314(66%)	—	—	682 (34%)	509 (75%)	LMM (BIA)
Van de Bool *et al*. 2016 (van de Bool *et al*. 2016)	Netherlands	Cross‐sectional	45	42–77	29 (64%)	—	I/II/III/IV (6/36/49/9)	14 (31%)	13 (92%)	LMM (DXA)
Lipovec *et al*. 2016^28^	Slovenia	Prospective observational	112	66 ± 8	74 (66%)	92 current smokers	I/II/III/IV (0/17/52/31)	61 (54%)	44 (72%)	LMM (DXA)
Borda *et al*. 2016^29^	Colombia	Cross‐sectional	334	71.1 ± 8.05	110 (33%)	—	—	28 (8%)	—	LMM (CC)
										LMS (HGS)
										LPP (3.4MGS)
Lee *et al*. 2016^30^	Korea	Cross‐sectional	858	—	—	—	—	286 (33%)	226 (79%)	LMM (DXA)
Pothirat *et al*. 2016^31^	Thailand	Cross‐sectional	121	—	—	121 former smokers	I/II/III/IV (26/25/10/39)	12 (10%)	—	LMM (BIA)
Maddock *et al*. 2016^32^	UK	Prospective cohort	816	69.8 ± 9.7	484 (59%)	49/620/146	—	101 (12%)	—	LMM (BIA)
										LMS (HGS)
										LPP (4MGS)
Hwang *et al*. 2017^13^	Korea	Cross‐sectional	777	63.9 ± 10.6	777 (100%)	0/185/592	I/II/III–IV (43/50/7)	41 (5.3%)	41 (100%)	LMM (DXA)
Limpawattana *et al*. 2017^33^	Thailand	Cross‐sectional	121	—	112 (92.6%)	7/104/10	—	29 (24%)	29 (100%)	LMM (DXA)
										LMS (HGS)
										LPP (6MWT)
Byun *et al*. 2017^1^	Korea	Cross‐sectional	80	68.4 ± 8.9	67 (83.8%)	—	I/II/III/IV (30/39/6/25)	20 (25%)	17 (83%)	LMM (BIA)
										LMS (HGS)
Limpawattana *et al*. 2017^34^	Thailand	Cross‐sectional	121	70 ± 9	112 (92.6%)	7/104/10	I/II/III/IV (26/57/17/0)	29 (24%)	29 (100%)	LMM (DXA)
										LMS (HGS)
										LPP (6MWT)
Lee *et al*. 2017^35^	Korea	Cross‐sectional	748	—	—	—	—	251 (34%)	203 (81%)	LMM (DXA)
Kneppers *et al*. 2017^36^	Slovenia	Prospective cohort	92	—	—	—	I/II/III/IV (3/24/50/23)	39 (42%)	29 (74%)	LMM (DXA)
Costa *et al*. 2017^37^	Brazil	Cross‐sectional	121	67.9 ± 8.6	56 (46%)	23 current smokers	—	13 (11%)		LMM (DXA) LPP (6MWT)
								6 (5%)		
								11 (9%)		
								15 (12%)		
Costa *et al*. 2018^38^	Brazil	Cross‐sectional	121	67.9 ± 8.6	56 (46%)	—	A/B/C/D (29/29/34/29)	15 (12%)	—	LMM (DXA)
										LPP (6MWT)
										LMS (HGS)
										LPP (4MGS)

3.4 MGS, 3.4 m gait speed; 4MGS, 4 m gait speed; 6MWT, 6 min walking test; BIA, bioelectrical impedance analysis; CC, calf circumference; DXA, dual‐energy X‐ray absorptiometry; HGS, handgrip strength; LMM, lower muscle mass; LMS, lower muscle strength; LPP, lower physical performance; SD, standard deviation.

### Methods used to assess sarcopenia

A summary of diagnostic criteria used to assess sarcopenia in the included studies is presented in *Table* 1. Measures of low muscle mass (LMM),^1^, ^2^, ^13^, ^21^, ^22^, ^23^, ^24^, ^25^, ^26^, ^27^, ^28^, ^29^, ^30^, ^31^, ^32^, ^33^, ^34^, ^35^, ^36^, ^37^, ^38^, ^39^, ^40^ low muscle strength (LMS),^1^, ^2^, ^29^, ^32^, ^33^, ^34^, ^39^ and low physical performance (LPP)^2^, ^29^, ^32^, ^33^, ^34^, ^37^, ^38^, ^39^ were used as the basis of diagnosis. Fourteen studies used LMM as the sole criteria to diagnose sarcopenia, while LMM was combined with LMS and/or LPP in nine studies.^1^, ^2^, ^29^, ^32^, ^33^, ^34^, ^37^, ^38^, ^39^ Those studies utilized different cut‐off points and methods to identify LMM, LMS, and LPP. Muscle mass was measured by dual‐energy X‐ray absorptiometry (sixteen studies),^13^, ^21^, ^22^, ^24^, ^25^, ^26^, ^28^, ^33^, ^34^, ^35^, ^36^, ^37^, ^38^, ^40^ bioelectrical impedance analysis (six studies),^1^, ^2^, ^23^, ^27^, ^31^, ^32^, ^39^ and calf circumference (one study).^29^ Muscle strength was measured via handgrip dynamometry (seven studies).^1^, ^2^, ^29^, ^32^, ^33^, ^34^, ^39^ Physical performance was measured via gait speed (four studies)^23^, ^29^, ^32^, ^39^ and 6 min walk test (6MWT) (four studies).^33^, ^34^, ^37^, ^38^ The different cut‐off thresholds used to define ‘positive’ responses to each test are presented in *Table* 2. Muscle mass, muscle strength, and physical performance were most commonly evaluated according to cut‐off thresholds recommended by the EWGSOP^7^ and the Asian Group of Sarcopenia.^15^ Comparisons between the main guidelines used to detect sarcopenia in individuals with COPD are available in *Table* S3.

**Table 2:** Criteria and cut‐off points used to detect sarcopenia in individuals with chronic obstructive pulmonary disease in the different studies

*Lower muscle mass*		References
DXA	1. EWGSOP^7^ Newman *et al*. 2003^41^ ASMI: <7.23 kg/m^2^ for men and <5.67 kg/m^2^ for women.	Van de Bool *et al*. 2015,^25^ Lipovec *et al*. 2016,^28^ Kneppers *et al*. 2017,^36^ and van de Bool *et al*.^40^
	2. EWGSOP^7^ Newman *et al*. 2003^41^ Residuals of linear regression on appendicular lean mass adjusted for fat as well as height. Men: −2.29, women: −1.73.	Costa *et al*. 2015^24^ and Costa *et al*. 2017^37^
	3. EWGSOP^7^ Baumgartner *et al*. 1998^42^ SMI: ≤7.26 kg/m^2^ for men and ≤5.45 kg/m^2^ for women.	Costa *et al*. 2015^24^ and Costa *et al*. 2017^37^
	4. AWGS^15^ ASMI: ≤7.0 kg/m^2^ for men and ≤5.4 kg/m^2^ for women.	Lee and Choi,^30^ Limpawattana *et al*.,^33^, ^34^ and Lee *et al*.^35^
	5. FNIH^43^ ALM/BMI: <0.789 for men and for < 0.512 women.	Costa *et al*. 2017^37^ and Costa *et al*. 2018^38^
	6. ASMMI: ≤ 2 standard deviations in a gender‐specific mean for a young reference group.	Byun *et al*.,^1^ Hwang *et al*.,^13^ Sergi *et al*.,^21^ Chung *et al*.,^26^ and van de Bool *et al*.^40^
	7. SMI:<1 standard deviations in a gender‐specific mean for a young reference group.	Koo *et al*.^22^
	8. Combination of criteria 2 and 3.	Costa *et al*. 2015^24^ and Costa *et al*. 2017^37^
BIA	1. EWGSOP^7^ Janssen *et al*. 2002^44^ SMI: ≤8.50 kg/m^2^ for men and ≤5.75 kg/m^2^ for women.	Jones *et al*.,^2^ Maddocks *et al*.,^32^ and de Blasio *et al*.^39^
	2. ATS^45^ BMI >21 and FFMI ≤16 kg/m^2^ for men or ≤15 kg/m^2^ for women.	Gologanu *et al*.^23^ and Pothirat *et al*.^31^
	3. Franssen *et al*. 2014^46^ Lower than the 10 percentile of the reference value for age, sex, and BMI specific.	Joppa *et al*.^27^
	4. ASMMI: ≤2 standard deviations in a gender‐specific mean for a young reference group.	Byun *et al*.^1^
CC	1. Calf circumference <31 cm.	Borda *et al*.^29^
*Lower muscle strength*		
HGS	1. EWGSOP^7^ Laurentani *et al*. 2003^47^ HGS: <30 kg for men and <20 kg for women.	Byun *et al*.,^1^ Jones *et al*.,^2^ Maddocks *et al*.,^32^ and de Blasio *et al*.^39^
	2. AWGS^15^ HGS: <26 kg for men and <18 kg for women.	Limpawattana *et al*.^33^, ^34^
	3. Lower the last quintile in specific population.	Borda *et al*.^29^
*Lower physical performance*		
4MGS	1. EWGSOP^7^ Laurentani *et al*. 2003^47^ GS: <0.8 m/s (both genders).	Jones *et al*.,^2^ Maddocks *et al*.,^32^ and de Blasio *et al*.^39^
3.4MGS	1. Lower the last quintile in specific population.	Borda *et al*.^29^
6MWT	1. AWGS^15^ Laurentani *et al*. 2003^47^ GS: <0.8 m/s (both genders).	Limpawattana *et al*.^33^, ^34^
	2. EWGSOP^7^ Laurentani *et al*. 2003^47^ GS: <0.8 m/s (both genders).	Costa *et al*. 2017^37^
	3. FNIH^43^ GS: <0.8 m/s (both genders).	Costa *et al*. 2018^38^

3.4 MGS, 3.4 m gait speed; 4MGS, 4 m gait speed; 6MWT, 6 min walking test; ASMI, appendicular skeletal muscle index; ATS, American Thoracic Society; AWGS, Asian Working Group for Sarcopenia; BIA, bioelectrical impedance analysis; BMI, body mass index; CC, calf circumference; DXA, dual‐energy X‐ray absorptiometry; EWGSOP, European Working Group on Sarcopenia in Older People; FNIH, The Foundation for the National Institutes of Health Sarcopenia Project; HGS, handgrip strength; SMI, skeletal muscle mass index.

### Sarcopenia prevalence

**Figure 2:**
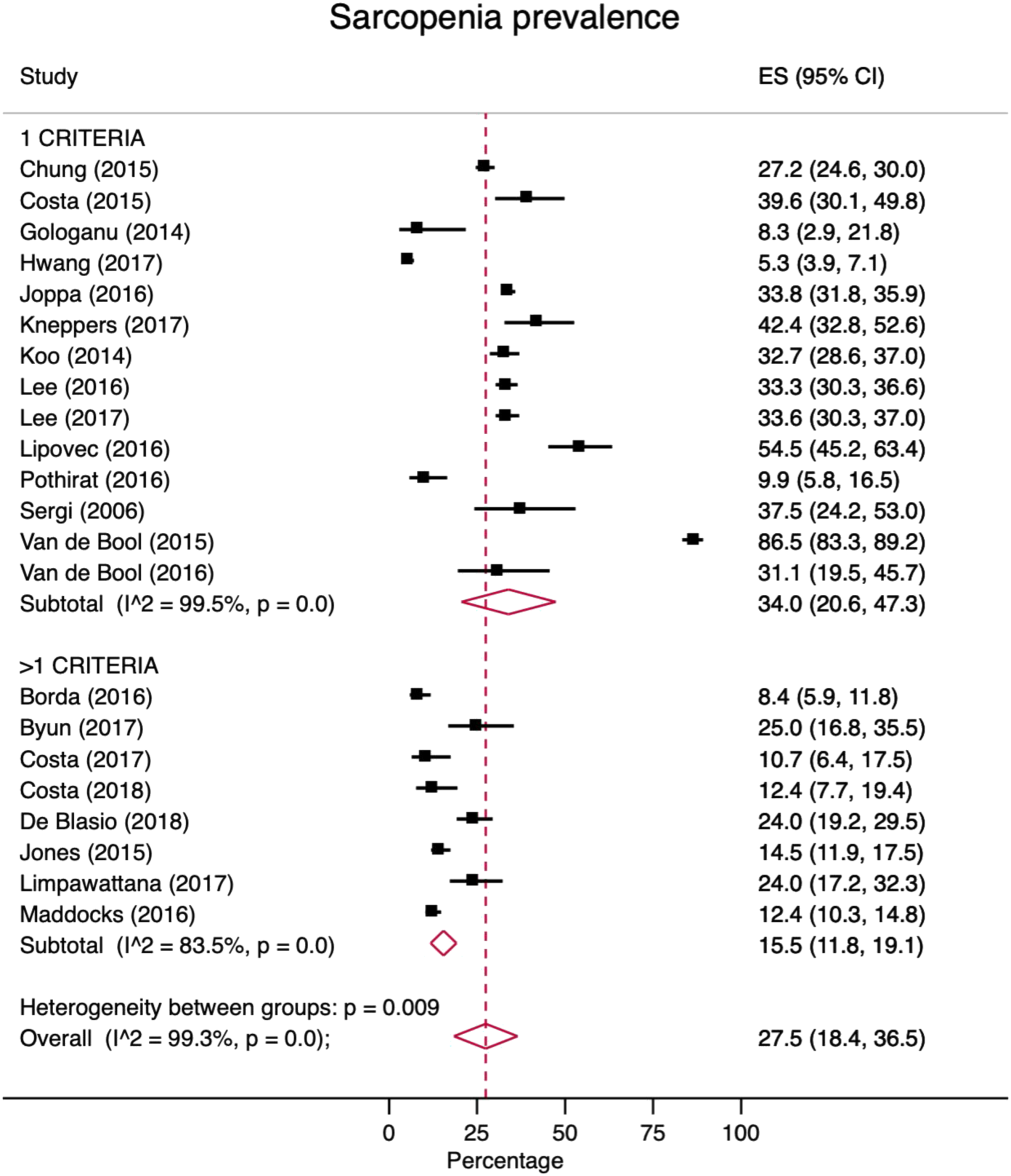
Prevalence of sarcopenia in chronic obstructive pulmonary disease according to different criteria. CI, confidence interval; ES, effect size (prevalence %); *I* ^2^, *I* ^2^ heterogeneity statistic. Random effects model used for analysis.

Data were available for meta‐analysis from 22 studies involving 9416 participants. The overall pooled prevalence estimate of sarcopenia in people with COPD was 27.5% (95%CI 18.4–36.5; *Figure* 2). These effect estimates were significantly higher in studies that used a single criterion [LMM; 34%, (95%CI 20.6–47.3)] than those that used >1 criteria [LMM + LMS and/or LPP; 15.5% (95%CI 11.8–19.1)]. The high statistical heterogeneity in this analysis (*I* ^2^ = 99.3%) meant that individual study weighting was uniform (range 4.1–4.7%). In the studies that provided data specific to gender, sarcopenia was found to be higher in men [41.0% (95%CI 26.2–55.9)] than in women [31.9% (95%CI 7.0–56.8)]; however, this difference was not statistically significant (*P* = 0.538) and gender did not predict effect size in meta‐regression (*Figures* S1–S2). In the studies that provided data specific to disease severity, sarcopenia was found to be significantly higher in patients with more severe disease [GOLD stages III–IV; 37.6% (95%CI 24.8–50.4)] than those with less severe disease [GOLD stages I–II; 19.1% (95%CI 10.2–28.0)], with test for between‐group differences (*P* = 0.020) with the proportion of participants having more severe disease being strongly predictive of effect sizes in meta‐regression with high explanatory power [regression coefficient 0.715 (95%CI 0.342–1.088), *P* = 0.006; adjusted *R* ^2^ = 90.1%] (*Figures* S3–S4).

### Impact of sarcopenia on clinical outcomes

**Figure 3:**
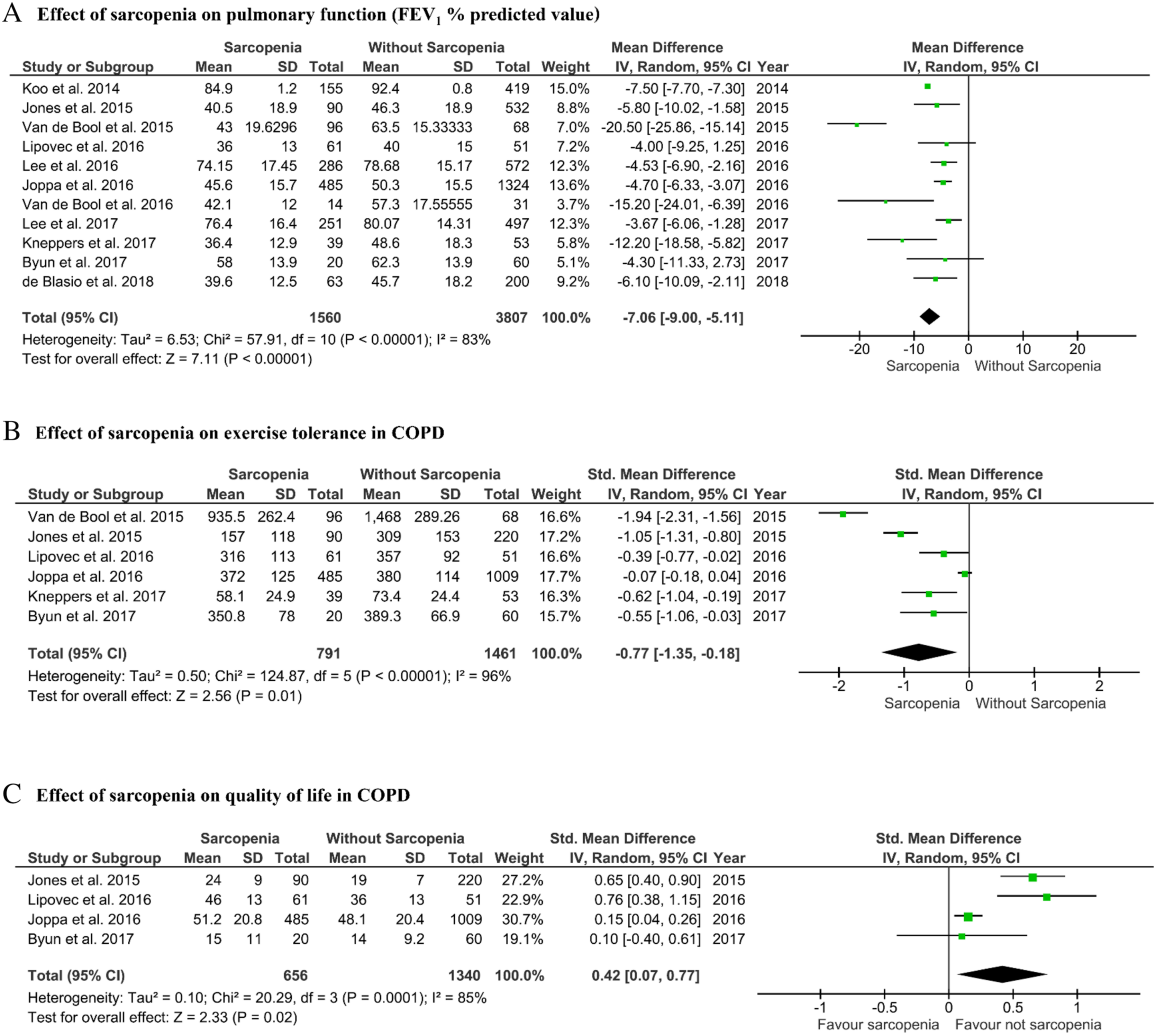
Clinical impact of sarcopenia in individuals with COPD. COPD, chronic obstructive pulmonary disease; *I* ^2^, *I* ^2^ heterogeneity statistic. Random effects model used for analysis.

Data from 11 studies involving 5367 participants were available for meta‐analysis of pulmonary function, showing that those with sarcopenia had, on average, poorer FEV_1_% predicted than those without sarcopenia [mean difference −7.07% (95%CI −9.03 to −5.11); *I* ^2^ = 83%, *Figure* 3A].

Data from six studies involving 2252 participants were available for outcomes related to exercise capacity. These were measured via the 6MWT,^1^, ^27^, ^28^ incremental shuttle walk test,^2^ and cardiopulmonary incremental cycle test.^25^, ^36^ Having sarcopenia was associated with poorer performance compared with those without sarcopenia [SMD −0.77 (95%CI −1.35 to −0.18); *I* ^2^ = 96%, *Figure* 3B].

Four studies involving 1996 participants reported data on quality of life via the COPD Assessment Test,^1^, ^2^ and St George's Respiratory Disease Questionnaire^2^, ^27^, ^28^ was included in the meta‐analysis. Having sarcopenia was associated with poorer quality of life [SMD 0.42 (95%CI 0.07–0.77); *I* ^2^ = 85%, *Figure* 3C]. Other studies not included in the meta‐analysis reported similar findings^33^, ^35^ (*Table* 3).

**Table 3:** Clinical impact of the sarcopenia in different variables in subjects with chronic obstructive pulmonary disease

Categories	Variables	Compared with individuals with COPD without sarcopenia	
		Sarcopenia (1 criterion)	Sarcopenia (>1 criterion)
Health‐related quality of life	EQ‐5D index (score)	Worse^30^, ^35^	
Physical function	SPPB (score)		Worse^2^
	5STS (s)		Worse^2^
	HGS (kg)		Worse^2^, ^39^
	QS (kg)	Worse ^21^	Worse^2^
	GS (m/s)		Reduction^2^, ^39^
Physical activity level	Time in moderate and high activity (min/day)	Worse^22^	Worse^2^
	Total energy expenditure (kcal/week)		Worse^2^
	Daily Steps (steps/day)		N.d.^2^
	Prevalence of physical inactivity	Worse^30^, ^35^	
Dyspnoea	MRC (score)	N.d.^21^	Worse^1^, ^2^
Risk of mortality	Prevalence in BODE quartile 3 or 4	Higher^24^	Higher^1^, ^2^, ^38^
Inflammation	CRP (mg/L)	Augmented^25^ /N.d^27^, ^28^	Augmented^39^
	Fibrinogen (mg/L)	N.d.^27^	
	IL‐6 (pg/mL)	N.d.^27^	Augmented^1^
	IL‐8 (pg/mL)	N.d.^27^	
	TNF‐α (pg/mL)	N.d.^27^	Augmented^1^

5STS, five‐repetition sit‐to‐stand test; 6MWT, 6 min walking test; BODE, body mass index, obstruction, dyspnoea, and exercise tolerance index; CAT, COPD Assessment Test; CRP, C‐reactive protein; EQ‐5D index, EuroQol five‐dimensional; GS, gait speed; HGS, handgrip strength; IL, interleukin; ISWT, incremental shuttle walk test; MRC, Medical Research Council; N.d., no significant difference; QS, quadriceps strength; SGRQ, St George's respiratory disease questionnaire; SPPB, short physical performance battery; TNF‐α, tumour necrosis factor‐alpha.

A summary of findings related to the remaining review outcomes is presented in *Table* 3; however, quantitative meta‐analysis was not possible due to lack of sufficient data. Compared with non‐sarcopenic individuals, those with sarcopenia had worse physical function (as measured by tests of balance, gait speed, strength, and general daily function),^2^, ^21^, ^39^ lower levels of daily physical activity,^2^, ^22^, ^30^, ^35^ increased levels of dyspnoea during daily activities,^1^, ^2^ and a heightened mortality risk, as measured via body mass index, obstruction, dyspnoea, and exercise tolerance (BODE) index.^1^, ^2^, ^38^ Sarcopenia was more prevalent in the fourth quartile of BODE, ranging from 25% to 63.6%.^1^, ^2^, ^38^ With respect to inflammatory biomarkers, C‐reactive protein, IL‐6, and tumour necrosis factor‐alpha were reported to be higher^1^, ^25^, ^39^ or not different^27^, ^28^ in subjects with sarcopenia compared with those without it. No differences were detected in levels of fibrinogen^27^ and IL‐8.^27^ No findings related to oxidative stress were reported in the included literature.

## Discussion

This systematic review and meta‐analysis offers unique insight into the clinical relevance of sarcopenia for people with COPD. It describes the prevalence of the condition and how this is impacted by use of different criteria, cut‐off thresholds and definitions, as well as rigorous examination of the effect of sarcopenia on important health outcomes related to pulmonary and physical function, quality of life, blood biomarkers, prognosis, and risk of mortality.

Two predominant strategies appear to be in use to classify sarcopenia in COPD: definitions based upon independent assessment of LMM^21^, ^22^, ^24^, ^27^, ^28^, ^30^, ^35^ and definitions that include both LMM and either LMS or LPP.^1^, ^2^, ^23^, ^29^, ^32^, ^33^, ^34^, ^37^, ^38^, ^39^ Use of LMM alone resulted in an estimated pooled prevalence of 34%, while LMM combined with LMS and/or physical function reduced this figure to 15.5%. Such variability has been previously reported in community‐dwelling older adults.^48^ Sarcopenia definition variability thus also likely explains some of the varied prevalence estimates in people with COPD. This relationship may not come as a surprise, as increasing the number of mandatory elements within a sarcopenia definition will inevitably reduce the incidence of detecting a ‘positive’ diagnosis. The trade‐off of doing so, however, is a likely improvement in diagnostic accuracy. This is a significant premise underpinning current international recommendations,^7^, ^19^, ^43^, ^49^ which sees sarcopenia defined as a geriatric syndrome^7^, ^15^, ^43^, ^49^ or disease^19^ characterized by both LMM and LPP, not just LMM.^50^, ^51^ Only nine of the included studies^1^, ^2^, ^29^, ^32^, ^33^, ^34^, ^37^, ^39^ implemented a definition of sarcopenia that would satisfy these new recommendations (*Table* 1). Our data suggest that some of the variability in prevalence estimates is likely attributable to disease severity, with every 1% increase in study sample having GOLD stages III–IV increasing sarcopenia prevalence by 0.7%. While this relationship was not unexpected based on previous research,^2^, ^32^ the high explanatory power (90.1%) in our meta‐regression was striking. Detailed reporting and/or stratification by disease severity in this patient group appears advisable to ensure that accurate conclusions are drawn from future studies seeking to advance our knowledge of the interplay between these two factors.

Recommendations advocate for dual‐energy X‐ray absorptiometry and bioelectrical impedance analysis as the preferred methods to evaluate LMM for the purpose of detecting sarcopenia, including evaluation of muscles of both the lower limb and the chest wall.^7^, ^15^, ^19^, ^41^, ^42^, ^43^, ^49^, ^52^, ^53^ These were commonly used within the studies included in this review (*Tables* 1 and 2). Despite this, we observed 12 different cut‐off points used to classify test results as normal or abnormal. The most commonly used criteria were those of Newman *et al*.^54^ and Baumgartner *et al.,* ^55^ which are also considered by the EWGSOP.^7^ Borda *et al*.^29^ measured muscle mass with calf circumference, which confers simplicity as a screening method for sarcopenia,^56^, ^57^ but it is not recommended.^7^, ^15^, ^19^, ^43^, ^49^ Similar advice is also available for the assessment of muscle strength (handgrip force) and physical performance (gait speed),^7^, ^15^ yet inconsistencies were again apparent. For example, gait speed was assessed using the 4 m gait speed^23^, ^29^, ^32^, ^39^ and the 6MWT.^33^, ^34^, ^37^, ^38^ While the same cut‐off was used to diagnose sarcopenia across both tests (<0.8 m/s), the two tests are vastly different. The 4 m gait speed is typically performed at usual walking speed across a 4 m distance (although variations also exist at different walk speeds and track lengths), while the 6MWT is typically performed on a 30 m walking track with participants encouraged to walk as far as they can (often faster than normal speed) in order to assess exercise tolerance.^58^ Deriving a measure of walking speed from the 6MWT [i.e. total distance (m) divided by 360 (s)] poses a significant risk of inaccurate interpretation. For example, it could not distinguish between people walking slowly and fast but stopping to rest during the test. The prevalence of sarcopenia in the studies that used this approach^33^, ^34^, ^37^, ^38^ may therefore have been underestimated. It is thus crucial that future research not only implement consistent tests to diagnose sarcopenia, but also adopt standardized cut‐off thresholds to facilitate accurate test interpretation.

Sarcopenia had a consistently negative impact on a range of COPD‐related clinical outcomes, including exercise capacity, balance, quadriceps, and handgrip strength, gait speed, and physical activity levels.^2^, ^21^, ^30^, ^35^, ^39^ It was also associated with increased symptom burden and poorer quality of life.^1^, ^2^, ^30^, ^35^ It is interesting that the two studies that measured dyspnoea (Medical Research Council scale)^1^, ^2^ classified sarcopenia according to physical function alone, as it raises the possibility that functional impairment may associate more strongly with dyspnoea than LMM.^21^ This also raises some challenging issues related to clinical management strategies. As associations do not imply causation or directionality, should interventions targeting improvement in health outcomes for people with COPD who have sarcopenia be directed towards mitigating the defining features of sarcopenia (e.g. muscle mass and physical performance) or their associated manifestations (e.g. low physical activity levels, poor balance, impaired lung function)? To our knowledge, the precise impact of sarcopenia (and its severity) upon intervention effectiveness targeting these other areas has received scant attention to date in COPD. Sarcopenia has, however, been highlighted as an important ‘treatable trait’ in adult respiratory medicine.^59^ One of the few studies to explore this area was conducted by Jones *et al*.^2^ who demonstrated that pulmonary rehabilitation, a comprehensive, multicomponent exercise‐based intervention, improved a range of clinical outcomes and reduced the incidence of sarcopenia in a cohort of patients with COPD. More research is clearly warranted to further validate the findings of Jones and colleagues, including the use of other recommended adjunctive therapies such as nutritional supplementation.^7^, ^60^, ^61^

We were not able to investigate actual mortality in those who had sarcopenia due to a lack of available evidence. However, it is plausible that sarcopenia might associate with increased mortality in this population, considering that it associated with poorer prognosis and a higher prevalence in patients with more severe lung disease (37.6% in GOLD stages III–IV compared with 19.1% in those with GOLD stages I–II). Leivseth *et al*.^62^ reported that people with GOLD stages III and IV disease severity had a more than sixfold increased risk of mortality in women and a more than double increased risk in men over 15 years of follow‐up. Heightened mortality risk was also observed in individuals with COPD evaluated via BODE,^1^, ^2^, ^24^, ^38^ which is a widely used, valid tool for predicting risk of death in COPD.^63^, ^64^ Costa *et al*.^24^ reported an increased prevalence of sarcopenia (odds ratio 3.89; 95%CI 1.21–12.46) in those with GOLD stages III and IV, and these quartiles are related with lower 4 year survival (18–57%).^63^ Sarcopenia also related to poorer quality of life and pulmonary and physical function, which are known factors associated with heightened mortality risk in COPD.^44^, ^45^ Sarcopenia has been associated with premature mortality in community‐dwelling older adults in a cohort study with 4425 older adults during a median 14.4 year follow‐up (hazard ratio 1.32; 95%CI 1.13–1.47).^46^ However, the lack of COPD‐specific data suggests that this remains an area in need of addressing in future research.

This systematic review has highlighted the clinical relevance of including measurements of muscle mass, muscle strength, and physical performance in individuals with COPD, as these variables clearly associate with sarcopenia, exacerbations, and poor prognosis.^47^, ^59^ The more widespread implementation of these measures in clinical practice could help identify patients with COPD at increased risk of future healthcare use related to exacerbations.^47^, ^65^ This is also an important priority from a public health economic perspective.^66^ In Europe, on average, the healthcare system spends €6725 per year per person (95%CI €6590–€6863) for each exacerbation of this disease.^67^ In older people, sarcopenia is consistently associated with increased risk of incident disability, falls, hospitalization, and mortality.^46^, ^68^, ^69^ Sarcopenia has been associated with increased breathlessness, exacerbation frequency, and frailty in individuals with COPD.^47^, ^70^, ^71^ Hospitalizations also hasten deconditioning and muscle weakness, thereby worsening the sarcopenic state.^47^, ^72^ Earlier identification of sarcopenia may therefore help direct preventive healthcare to positively impact upon its healthcare burden.

We were unable to demonstrate a clear relationship between sarcopenia and inflammatory biomarkers across the included studies. Some authors^27^, ^28^, ^39^ reported no differences between sarcopenic and non‐sarcopenic patients with COPD, while Byun *et al*.^1^ and Van de Bool *et al*.^25^ observed higher levels of C‐reactive protein, IL‐6, and tumour necrosis factor‐alpha. No studies evaluated the effect of sarcopenia on oxidative stress, despite convincing evidence of pathophysiological changes occurring in the COPD literature^73^, ^74^, ^75^ and known associations between sarcopenia, oxidative stress,^76^, ^77^ inflammation,^1^ and age‐related alterations in muscle morphology.^76^, ^78^, ^79^, ^80^ This would appear a valuable area for future research.

As with all studies, the findings from the present review are not without some limitations. Due to the significant heterogeneity between studies in terms of factors such as sarcopenia definitions, participant characteristics, and diagnostic cut‐offs, the opportunity for meta‐analysis was limited for some outcomes and clear interpretation of the clinical implications of some results was challenging. This review was unable to elucidate the direct relationship between sarcopenia and mortality due to a lack of data. This was not surprising due to the prolonged periods of follow‐up required to observe such outcomes in cohorts of patients who would otherwise not typically have been at risk of imminent death. However, our observed association between sarcopenia and mortality risk (assessed via BODE) is noteworthy. While not a pre‐specified focus of our review, we also feel that the lack of direct evidence highlighting the clinical impact of sarcopenia on healthcare expenditure represents an area to address in future studies. Additionally, despite this review including studies from four different continents (Asia, Europe, North America, and South America), data regarding participant race were not available, which limits its potential applicability to specific patient subgroups. In addition, it was not considered the impact of differing sarcopenia subtypes (e.g. sarcopenic obesity, severe sarcopenia), despite their clinical relevance due to a lack of suitable data. This might have plausibly explained some of the observed variability in clinical outcome data. We also synthesized prevalence data via meta‐analysis in contrast to our registered protocol. This was altered in light of access to appropriate statistical software to conduct this analysis while still allowing readers to identify the raw proportions of individual studies (as stated in the protocol) in *Figure* 2. The overall pooled effect from the present meta‐analysis (27.5%) compared favourably against the protocol‐based method utilizing median estimates from individual studies (26.1%).

In conclusion, sarcopenia is a clinically important condition that is prevalent within a substantial proportion of patients with COPD. Diagnostic accuracy appears sensitive to the criteria, test methods, and cut‐offs used to detect the individual components, as well as markers of disease severity. Considering the negative impact of sarcopenia upon health outcomes, there may be merit in future strategies targeting early identification of sarcopenia in the clinical assessment of people with COPD to ultimately improve management strategies aiming to mitigate its impact upon individuals' lives.

## Author contributions

W.S.L. and A.A.M. had full access to all of the data in the study and takes responsibility for the integrity of the data and the accuracy of the data analysis, C.O. and S.P. contributed substantially to statistical analysis and interpretation of the results, and G.D. and V.S.P. contributed with the study design and writing of the manuscript. The authors of this manuscript certify that they comply with the ethical guidelines for publishing in the *Journal of Cachexia, Sarcopenia and Muscle*.^81^

## Conflict of interest

The authors have disclosed no conflicts of interest. We declare no financial support or relationships that may pose conflict of interest. This work has not been published anywhere.

## Funding

This study was financed in part by the Coordenação de Aperfeiçoamento de Pessoal de Nível Superior – Brasil (CAPES) – Finance Code 001. WASL is supported by CAPES ‐ Brasil. VSP is supported by the Conselho Nacional de Desenvolvimento Científico e Tecnológico (CNPq) ‐ Brasil.

## Online supplementary material

The supplementary figures and tables can be found in the Supporting Information section of the online article.

## Supporting information

Table S1: Search strategy in each database (Supplementary data)
Table S2: Quality analysis (Supplementary data)
Table S3: Different cut‐off points used to identify Sarcopenia.
Figure S1: Prevalence of sarcopenia by gender.
Figure S2. Meta‐regression of effect of gender (percent male) on sarcopenia prevalence.
Figure S3. Prevalence of sarcopenia, by COPD disease severity.
Figure S4. Meta‐regression of effect of disease severity (GOLD stages III‐IV) on sarcopenia prevalence.
