# Supplementary material for: Diagnosis, prevalence, and clinical impact of sarcopenia in COPD: a systematic review and meta‐analysis
Source: J Cachexia Sarcopenia Muscle. 2020 Aug 30;11(5):1164–76. doi: 10.1002/jcsm.12600 (PMC7567149; doi:10.1002/jcsm.12600)
Supplement: Supplementary file 1 — Table S1: Search strategy in each database (Supplementary data) Table S2: Quality analysis (Supplementary data) Table S3: Different cut‐off points used to identify Sarcopenia. Figure S1: Prevalence of sarcopenia by gender. Figure S2. Meta‐regression of effect of gender (percent male) on sarcopenia prevalence. Figure S3. Prevalence of sarcopenia, by COPD disease severity. Figure S4. Meta‐regression of effect of disease severity (GOLD stages III‐IV) on sarcopenia prevalence. [file JCSM-11-1164-s001.docx]

**Supplemental Material**

| **Table S1: Search strategy in each database (Supplementary data)** | |
| --- | --- |
| **Pubmed** | |
| Search #3  (74 articles) | #1 [All Fields] AND #2[All Fields] |
| Search #2  (5725 articles) | (sarcopenia[MeSH Terms]) OR Sarcope*[Title/Abstract] |
| Search #1  (62641 articles) | Pulmonary Disease, Chronic Obstructive [Mesh Terms] OR COPD [Text Word] |
| **LILACS** | |
| Search #3  (103 articles) | (tw:((tw:(DPOC)) OR (tw:(COPD)) OR (tw:(Pulmonary Disease, Chronic Obstructive )))) AND (tw:(Sarcopeni*)) |
| Search #2  (5,935 articles) | (tw:(Sarcopeni*)) |
| Search #1  (69,143 articles) | (tw:(DPOC)) OR (tw:(COPD)) OR (tw:(Pulmonary Disease, Chronic Obstructive )) |
| **EMBASE** | |
| Search #6  (116 articles) | #4 AND #5 |
| Search #5  (7,867 articles) | 'sarcopeni*':ab,ti |
| Search #4  (73,473articles) | 'copd':ab,ti OR 'chronic obstructive lung disease':ab,ti |
| Search #3  (228 articles) | #1 AND #2 |
| Search #1  (10,753 articles) | 'sarcopeni*' |
| Search #1  (109,757 articles) | 'copd'/exp OR 'chronic obstructive lung disease'/exp |
| **Cochrane Library** | |
| Search #3  (10 articles) | #1 AND #2 |
| Search #2  (456 articles) | "sarcopenia":ti,ab,kw or sarcopenic (Word variations have been searched) |
| Search #1  (14507 articles) | "COPD":ti,ab,kw or Pulmonary Disease, Chronic Obstructive (Word variations have been searched) |
| **Scielo** | |
| Search #8  (1 article) | #6 AND #7 |
| Search #7  (125) | (ab:(Sarcopeni*)) |
| Search 6  (1097) | #1 OR #2 OR #3 OR #4 OR #5 |
| Search #5  (337) | (ab:(Enfermedad Pulmonar Obstructiva Crónica )) |
| Search # 4  (320) | (ab:(Doença Pulmonar Obstrutiva Crônica)) |
| Search # 3  (327) | (ab:(DPOC)) |
| Search # 2  (707) | (ab:(Pulmonary Disease, Chronic Obstructive )) |
| Search # 1  (702) | (ab:(COPD)) |

| **Table S2: Quality analysis (Supplementary data)** | | | |
| --- | --- | --- | --- |
| Instrument: NIH Quality Assessment Tool for Observational Cohort and Cross-Sectional Studies | | | |
| Author: Sergi *et alet al*.2006 | | | |
| Criteria | **Yes** | **No** | **Other (CD, NR, NA)*** |
| 1. Was the research question or objective in this paper clearly stated? | x |  |  |
| 1. Was the study population clearly specified and defined? | x |  |  |
| 1. Was the participation rate of eligible persons at least 50%? |  | x |  |
| 1. Were all the subjects selected or recruited from the same or similar populations (including the same time period)? Were inclusion and exclusion criteria for being in the study prespecified and applied uniformly to all participants? | x |  |  |
| 1. Was a sample size justification, power description, or variance and effect estimates provided? |  | x |  |
| 1. For the analyses in this paper, were the exposure(s) of interest measured prior to the outcome(s) being measured? |  |  | x |
| 1. Was the timeframe sufficient so that one could reasonably expect to see an association between exposure and outcome if it existed? |  |  | x |
| 1. For exposures that can vary in amount or level, did the study examine different levels of the exposure as related to the outcome (e.g., categories of exposure, or exposure measured as continuous variable)? | x |  |  |
| 1. Were the exposure measures (independent variables) clearly defined, valid, reliable, and implemented consistently across all study participants? | x |  |  |
| 1. Was the exposure(s) assessed more than once over time? |  |  | x |
| 1. Were the outcome measures (dependent variables) clearly defined, valid, reliable, and implemented consistently across all study participants? | x |  |  |
| 1. Were the outcome assessors blinded to the exposure status of participants? |  |  | x |
| 1. Was loss to follow-up after baseline 20% or less? |  |  | x |
| 1. Were key potential confounding variables measured and adjusted statistically for their impact on the relationship between exposure(s) and outcome(s)? | x |  |  |

| Author: Koo *et al*.2014 | | | |
| --- | --- | --- | --- |
| Criteria | **Yes** | **No** | **Other (CD, NR, NA)*** |
| 1. Was the research question or objective in this paper clearly stated? | x |  |  |
| 1. Was the study population clearly specified and defined? | x |  |  |
| 1. Was the participation rate of eligible persons at least 50%? |  |  | x |
| 1. Were all the subjects selected or recruited from the same or similar populations (including the same time period)? Were inclusion and exclusion criteria for being in the study prespecified and applied uniformly to all participants? | x |  |  |
| 1. Was a sample size justification, power description, or variance and effect estimates provided? |  | x |  |
| 1. For the analyses in this paper, were the exposure(s) of interest measured prior to the outcome(s) being measured? |  |  | x |
| 1. Was the timeframe sufficient so that one could reasonably expect to see an association between exposure and outcome if it existed? | x |  |  |
| 1. For exposures that can vary in amount or level, did the study examine different levels of the exposure as related to the outcome (e.g., categories of exposure, or exposure measured as continuous variable)? | x |  |  |
| 1. Were the exposure measures (independent variables) clearly defined, valid, reliable, and implemented consistently across all study participants? | x |  |  |
| 1. Was the exposure(s) assessed more than once over time? |  |  | x |
| 1. Were the outcome measures (dependent variables) clearly defined, valid, reliable, and implemented consistently across all study participants? | x |  |  |
| 1. Were the outcome assessors blinded to the exposure status of participants? |  |  | x |
| 1. Was loss to follow-up after baseline 20% or less? |  |  | x |
| 1. Were key potential confounding variables measured and adjusted statistically for their impact on the relationship between exposure(s) and outcome(s)? | x |  |  |

| Author: Gologanu *et al*. 2014 | | | |
| --- | --- | --- | --- |
| Criteria | **Yes** | **No** | **Other (CD, NR, NA)*** |
| 1. Was the research question or objective in this paper clearly stated? | x |  |  |
| 1. Was the study population clearly specified and defined? | x |  |  |
| 1. Was the participation rate of eligible persons at least 50%? |  |  | x |
| 1. Were all the subjects selected or recruited from the same or similar populations (including the same time period)? Were inclusion and exclusion criteria for being in the study prespecified and applied uniformly to all participants? | x |  |  |
| 1. Was a sample size justification, power description, or variance and effect estimates provided? |  | x |  |
| 1. For the analyses in this paper, were the exposure(s) of interest measured prior to the outcome(s) being measured? |  |  | x |
| 1. Was the timeframe sufficient so that one could reasonably expect to see an association between exposure and outcome if it existed? |  |  | x |
| 1. For exposures that can vary in amount or level, did the study examine different levels of the exposure as related to the outcome (e.g., categories of exposure, or exposure measured as continuous variable)? | x |  |  |
| 1. Were the exposure measures (independent variables) clearly defined, valid, reliable, and implemented consistently across all study participants? | x |  |  |
| 1. Was the exposure(s) assessed more than once over time? |  |  | x |
| 1. Were the outcome measures (dependent variables) clearly defined, valid, reliable, and implemented consistently across all study participants? | x |  |  |
| 1. Were the outcome assessors blinded to the exposure status of participants? |  |  | x |
| 1. Was loss to follow-up after baseline 20% or less? |  |  | x |
| 1. Were key potential confounding variables measured and adjusted statistically for their impact on the relationship between exposure(s) and outcome(s)? | x |  |  |
| Instrument: JBI Critical Appraisal Checklist for Quasi-Experimental Studies (non-randomized experimental studies) | | | |
| Author: Gologanu *et al*. 2014 |  |  |  |
| **Other (CD, NR, NA)*** | **Other (CD, NR, NA)*** | **Other (CD, NR, NA)*** | **Other (CD, NR, NA)*** |
| 1. Is it clear in the study what is the ‘cause’ and what is the ‘effect’ (i.e. there is no confusion about which variable comes first)? | x |  |  |
| 1. Were the participants included in any comparisons similar? | x |  |  |
| 1. Were the participants included in any comparisons receiving similar treatment/care, other than the exposure or intervention of interest? | x |  |  |
| 1. Was there a control group? | x |  |  |
| 1. Were there multiple measurements of the outcome both pre and post the intervention/exposure? | x |  |  |
| 1. Was follow up complete and if not, were differences between groups in terms of their follow up adequately described and analyzed? |  |  |  |
| 1. Were the outcomes of participants included in any comparisons measured in the same way? | x |  |  |
| 1. Were outcomes measured in a reliable way? | x |  |  |
| 1. Was appropriate statistical analysis used? | **x** |  |  |
| Instrument: NIH Quality Assessment Tool for Observational Cohort and Cross-Sectional Studies | | | |
| Author: Costa *et al*. 2015 | | | |
| Criteria | **Yes** | **No** | **Other (CD, NR, NA)*** |
| 1. Was the research question or objective in this paper clearly stated? | x |  |  |
| 1. Was the study population clearly specified and defined? | x |  |  |
| 1. Was the participation rate of eligible persons at least 50%? | x |  |  |
| 1. Were all the subjects selected or recruited from the same or similar populations (including the same time period)? Were inclusion and exclusion criteria for being in the study prespecified and applied uniformly to all participants? | x |  |  |
| 1. Was a sample size justification, power description, or variance and effect estimates provided? |  | x |  |
| 1. For the analyses in this paper, were the exposure(s) of interest measured prior to the outcome(s) being measured? | x |  |  |
| 1. Was the timeframe sufficient so that one could reasonably expect to see an association between exposure and outcome if it existed? | x |  |  |
| 1. For exposures that can vary in amount or level, did the study examine different levels of the exposure as related to the outcome (e.g., categories of exposure, or exposure measured as continuous variable)? | x |  |  |
| 1. Were the exposure measures (independent variables) clearly defined, valid, reliable, and implemented consistently across all study participants? | x |  |  |
| 1. Was the exposure(s) assessed more than once over time? |  | x |  |
| 1. Were the outcome measures (dependent variables) clearly defined, valid, reliable, and implemented consistently across all study participants? | x |  |  |
| 1. Were the outcome assessors blinded to the exposure status of participants? |  |  | x |
| 1. Was loss to follow-up after baseline 20% or less? |  |  | x |
| 1. Were key potential confounding variables measured and adjusted statistically for their impact on the relationship between exposure(s) and outcome(s)? |  | x |  |
| Author: Van de bool *et al*. 2015 | | | |
| Criteria | **Yes** | **No** | **Other (CD, NR, NA)*** |
| 1. Was the research question or objective in this paper clearly stated? | x |  |  |
| 1. Was the study population clearly specified and defined? | x |  |  |
| 1. Was the participation rate of eligible persons at least 50%? |  |  | x |
| 1. Were all the subjects selected or recruited from the same or similar populations (including the same time period)? Were inclusion and exclusion criteria for being in the study prespecified and applied uniformly to all participants? | x |  |  |
| 1. Was a sample size justification, power description, or variance and effect estimates provided? |  | x |  |
| 1. For the analyses in this paper, were the exposure(s) of interest measured prior to the outcome(s) being measured? |  |  | x |
| 1. Was the timeframe sufficient so that one could reasonably expect to see an association between exposure and outcome if it existed? | x |  |  |
| 1. For exposures that can vary in amount or level, did the study examine different levels of the exposure as related to the outcome (e.g., categories of exposure, or exposure measured as continuous variable)? | x |  |  |
| 1. Were the exposure measures (independent variables) clearly defined, valid, reliable, and implemented consistently across all study participants? | x |  |  |
| 1. Was the exposure(s) assessed more than once over time? |  |  | x |
| 1. Were the outcome measures (dependent variables) clearly defined, valid, reliable, and implemented consistently across all study participants? | x |  |  |
| 1. Were the outcome assessors blinded to the exposure status of participants? |  |  | x |
| 1. Was loss to follow-up after baseline 20% or less? |  |  | x |
| 1. Were key potential confounding variables measured and adjusted statistically for their impact on the relationship between exposure(s) and outcome(s)? | x |  |  |
| Author: Chung *et al* 2015. | | | |
| Criteria | **Yes** | **No** | **Other (CD, NR, NA)*** |
| 1. Was the research question or objective in this paper clearly stated? | x |  |  |
| 1. Was the study population clearly specified and defined? | x |  |  |
| 1. Was the participation rate of eligible persons at least 50%? |  | x |  |
| 1. Were all the subjects selected or recruited from the same or similar populations (including the same time period)? Were inclusion and exclusion criteria for being in the study prespecified and applied uniformly to all participants? | x |  |  |
| 1. Was a sample size justification, power description, or variance and effect estimates provided? |  | x |  |
| 1. For the analyses in this paper, were the exposure(s) of interest measured prior to the outcome(s) being measured? |  |  | x |
| 1. Was the timeframe sufficient so that one could reasonably expect to see an association between exposure and outcome if it existed? | x |  |  |
| 1. For exposures that can vary in amount or level, did the study examine different levels of the exposure as related to the outcome (e.g., categories of exposure, or exposure measured as continuous variable)? | x |  |  |
| 1. Were the exposure measures (independent variables) clearly defined, valid, reliable, and implemented consistently across all study participants? | x |  |  |
| 1. Was the exposure(s) assessed more than once over time? |  |  | x |
| 1. Were the outcome measures (dependent variables) clearly defined, valid, reliable, and implemented consistently across all study participants? | x |  |  |
| 1. Were the outcome assessors blinded to the exposure status of participants? |  |  | x |
| 1. Was loss to follow-up after baseline 20% or less? |  |  | x |
| 1. Were key potential confounding variables measured and adjusted statistically for their impact on the relationship between exposure(s) and outcome(s)? | x |  |  |
| Author: Joppa *et al* 2016 | | | |
| Criteria | **Yes** | **No** | **Other (CD, NR, NA)*** |
| 1. Was the research question or objective in this paper clearly stated? | x |  |  |
| 1. Was the study population clearly specified and defined? | x |  |  |
| 1. Was the participation rate of eligible persons at least 50%? | x |  |  |
| 1. Were all the subjects selected or recruited from the same or similar populations (including the same time period)? Were inclusion and exclusion criteria for being in the study prespecified and applied uniformly to all participants? | x |  |  |
| 1. Was a sample size justification, power description, or variance and effect estimates provided? |  | x |  |
| 1. For the analyses in this paper, were the exposure(s) of interest measured prior to the outcome(s) being measured? | x |  |  |
| 1. Was the timeframe sufficient so that one could reasonably expect to see an association between exposure and outcome if it existed? | x |  |  |
| 1. For exposures that can vary in amount or level, did the study examine different levels of the exposure as related to the outcome (e.g., categories of exposure, or exposure measured as continuous variable)? | x |  |  |
| 1. Were the exposure measures (independent variables) clearly defined, valid, reliable, and implemented consistently across all study participants? | x |  |  |
| 1. Was the exposure(s) assessed more than once over time? |  | x |  |
| 1. Were the outcome measures (dependent variables) clearly defined, valid, reliable, and implemented consistently across all study participants? | x |  |  |
| 1. Were the outcome assessors blinded to the exposure status of participants? |  |  | x |
| 1. Was loss to follow-up after baseline 20% or less? |  |  | x |
| 1. Were key potential confounding variables measured and adjusted statistically for their impact on the relationship between exposure(s) and outcome(s)? | x |  |  |
| Author: Van de Bool *et al*. 2016 | | | |
| Criteria | **Yes** | **No** | **Other (CD, NR, NA)*** |
| 1. Was the research question or objective in this paper clearly stated? | x |  |  |
| 1. Was the study population clearly specified and defined? | x |  |  |
| 1. Was the participation rate of eligible persons at least 50%? |  |  | x |
| 1. Were all the subjects selected or recruited from the same or similar populations (including the same time period)? Were inclusion and exclusion criteria for being in the study prespecified and applied uniformly to all participants? | x |  |  |
| 1. Was a sample size justification, power description, or variance and effect estimates provided? |  | x |  |
| 1. For the analyses in this paper, were the exposure(s) of interest measured prior to the outcome(s) being measured? |  |  | x |
| 1. Was the timeframe sufficient so that one could reasonably expect to see an association between exposure and outcome if it existed? | x |  |  |
| 1. For exposures that can vary in amount or level, did the study examine different levels of the exposure as related to the outcome (e.g., categories of exposure, or exposure measured as continuous variable)? | x |  |  |
| 1. Were the exposure measures (independent variables) clearly defined, valid, reliable, and implemented consistently across all study participants? | x |  |  |
| 1. Was the exposure(s) assessed more than once over time? |  | x |  |
| 1. Were the outcome measures (dependent variables) clearly defined, valid, reliable, and implemented consistently across all study participants? | x |  |  |
| 1. Were the outcome assessors blinded to the exposure status of participants? |  |  | x |
| 1. Was loss to follow-up after baseline 20% or less? |  |  | x |
| 1. Were key potential confounding variables measured and adjusted statistically for their impact on the relationship between exposure(s) and outcome(s)? |  | x |  |
| Instrument: JBI Critical Appraisal Checklist for Quasi-Experimental Studies (non-randomized experimental studies) | | | |
| Author: Lipovec *et al*.2016 | | | |
| Criteria | **Yes** | **No** | **Other (CD, NR, NA)*** |
| 1. Is it clear in the study what is the ‘cause’ and what is the ‘effect’ (i.e. there is no confusion about which variable comes first)? | x |  |  |
| 1. Were the participants included in any comparisons similar? | x |  |  |
| 1. Were the participants included in any comparisons receiving similar treatment/care, other than the exposure or intervention of interest? | x |  |  |
| 1. Was there a control group? |  | x |  |
| 1. Were there multiple measurements of the outcome both pre and post the intervention/exposure? | x |  |  |
| 1. Was follow up complete and if not, were differences between groups in terms of their follow up adequately described and analyzed? |  |  |  |
| 1. Were the outcomes of participants included in any comparisons measured in the same way? | x |  |  |
| 1. Were outcomes measured in a reliable way? | x |  |  |
| 1. Was appropriate statistical analysis used? | x |  |  |
| Instrument: NIH Quality Assessment Tool for Observational Cohort and Cross-Sectional Studies | | | |
| Author: Borda *et al*.2016 | | | |
| Criteria | **Yes** | **No** | **Other (CD, NR, NA)*** |
| 1. Was the research question or objective in this paper clearly stated? | x |  |  |
| 1. Was the study population clearly specified and defined? | x |  |  |
| 1. Was the participation rate of eligible persons at least 50%? | x |  |  |
| 1. Were all the subjects selected or recruited from the same or similar populations (including the same time period)? Were inclusion and exclusion criteria for being in the study prespecified and applied uniformly to all participants? | x |  |  |
| 1. Was a sample size justification, power description, or variance and effect estimates provided? |  | x |  |
| 1. For the analyses in this paper, were the exposure(s) of interest measured prior to the outcome(s) being measured? |  |  | x |
| 1. Was the timeframe sufficient so that one could reasonably expect to see an association between exposure and outcome if it existed? | x |  |  |
| 1. For exposures that can vary in amount or level, did the study examine different levels of the exposure as related to the outcome (e.g., categories of exposure, or exposure measured as continuous variable)? | x |  |  |
| 1. Were the exposure measures (independent variables) clearly defined, valid, reliable, and implemented consistently across all study participants? | x |  |  |
| 1. Was the exposure(s) assessed more than once over time? |  |  | x |
| 1. Were the outcome measures (dependent variables) clearly defined, valid, reliable, and implemented consistently across all study participants? | x |  |  |
| 1. Were the outcome assessors blinded to the exposure status of participants? |  |  | x |
| 1. Was loss to follow-up after baseline 20% or less? |  |  | x |
| 1. Were key potential confounding variables measured and adjusted statistically for their impact on the relationship between exposure(s) and outcome(s)? | x |  |  |
| Author: Lee *et al*. 2016 | | | |
| Criteria | **Yes** | **No** | **Other (CD, NR, NA)*** |
| 1. Was the research question or objective in this paper clearly stated? | x |  |  |
| 1. Was the study population clearly specified and defined? | x |  |  |
| 1. Was the participation rate of eligible persons at least 50%? |  | x |  |
| 1. Were all the subjects selected or recruited from the same or similar populations (including the same time period)? Were inclusion and exclusion criteria for being in the study prespecified and applied uniformly to all participants? | x |  |  |
| 1. Was a sample size justification, power description, or variance and effect estimates provided? |  | x |  |
| 1. For the analyses in this paper, were the exposure(s) of interest measured prior to the outcome(s) being measured? |  |  | x |
| 1. Was the timeframe sufficient so that one could reasonably expect to see an association between exposure and outcome if it existed? | x |  |  |
| 1. For exposures that can vary in amount or level, did the study examine different levels of the exposure as related to the outcome (e.g., categories of exposure, or exposure measured as continuous variable)? | x |  |  |
| 1. Were the exposure measures (independent variables) clearly defined, valid, reliable, and implemented consistently across all study participants? | x |  |  |
| 1. Was the exposure(s) assessed more than once over time? |  |  | x |
| 1. Were the outcome measures (dependent variables) clearly defined, valid, reliable, and implemented consistently across all study participants? | x |  |  |
| 1. Were the outcome assessors blinded to the exposure status of participants? |  |  | x |
| 1. Was loss to follow-up after baseline 20% or less? |  |  | x |
| 1. Were key potential confounding variables measured and adjusted statistically for their impact on the relationship between exposure(s) and outcome(s)? | x |  |  |
| Author: Pothirat *et al*. 2016 | | | |
| Criteria | **Yes** | **No** | **Other (CD, NR, NA)*** |
| 1. Was the research question or objective in this paper clearly stated? | x |  |  |
| 1. Was the study population clearly specified and defined? | x |  |  |
| 1. Was the participation rate of eligible persons at least 50%? |  |  | x |
| 1. Were all the subjects selected or recruited from the same or similar populations (including the same time period)? Were inclusion and exclusion criteria for being in the study prespecified and applied uniformly to all participants? | x |  |  |
| 1. Was a sample size justification, power description, or variance and effect estimates provided? |  | x |  |
| 1. For the analyses in this paper, were the exposure(s) of interest measured prior to the outcome(s) being measured? |  |  | x |
| 1. Was the timeframe sufficient so that one could reasonably expect to see an association between exposure and outcome if it existed? | x |  |  |
| 1. For exposures that can vary in amount or level, did the study examine different levels of the exposure as related to the outcome (e.g., categories of exposure, or exposure measured as continuous variable)? | x |  |  |
| 1. Were the exposure measures (independent variables) clearly defined, valid, reliable, and implemented consistently across all study participants? | x |  |  |
| 1. Was the exposure(s) assessed more than once over time? |  |  | x |
| 1. Were the outcome measures (dependent variables) clearly defined, valid, reliable, and implemented consistently across all study participants? | x |  |  |
| 1. Were the outcome assessors blinded to the exposure status of participants? |  |  | x |
| 1. Was loss to follow-up after baseline 20% or less? |  |  | x |
| 1. Were key potential confounding variables measured and adjusted statistically for their impact on the relationship between exposure(s) and outcome(s)? | x |  |  |
| Author: Maddock *et al*. 2016 | | | |
| Criteria | **Yes** | **No** | **Other (CD, NR, NA)*** |
| 1. Was the research question or objective in this paper clearly stated? | x |  |  |
| 1. Was the study population clearly specified and defined? | X |  |  |
| 1. Was the participation rate of eligible persons at least 50%? |  |  | X |
| 1. Were all the subjects selected or recruited from the same or similar populations (including the same time period)? Were inclusion and exclusion criteria for being in the study prespecified and applied uniformly to all participants? | X |  |  |
| 1. Was a sample size justification, power description, or variance and effect estimates provided? |  | X |  |
| 1. For the analyses in this paper, were the exposure(s) of interest measured prior to the outcome(s) being measured? | X |  |  |
| 1. Was the timeframe sufficient so that one could reasonably expect to see an association between exposure and outcome if it existed? |  | X |  |
| 1. For exposures that can vary in amount or level, did the study examine different levels of the exposure as related to the outcome (e.g., categories of exposure, or exposure measured as continuous variable)? | X |  |  |
| 1. Were the exposure measures (independent variables) clearly defined, valid, reliable, and implemented consistently across all study participants? | X |  |  |
| 1. Was the exposure(s) assessed more than once over time? | X |  |  |
| 1. Were the outcome measures (dependent variables) clearly defined, valid, reliable, and implemented consistently across all study participants? | X |  |  |
| 1. Were the outcome assessors blinded to the exposure status of participants? |  | X |  |
| 1. Was loss to follow-up after baseline 20% or less? | X |  |  |
| 1. Were key potential confounding variables measured and adjusted statistically for their impact on the relationship between exposure(s) and outcome(s)? | X |  |  |
| Author: Hwang *et al*. 2017 | | | |
| Criteria | **Yes** | **No** | **Other (CD, NR, NA)*** |
| 1. Was the research question or objective in this paper clearly stated? | x |  |  |
| 1. Was the study population clearly specified and defined? | x |  |  |
| 1. Was the participation rate of eligible persons at least 50%? |  | x |  |
| 1. Were all the subjects selected or recruited from the same or similar populations (including the same time period)? Were inclusion and exclusion criteria for being in the study prespecified and applied uniformly to all participants? | x |  |  |
| 1. Was a sample size justification, power description, or variance and effect estimates provided? |  | x |  |
| 1. For the analyses in this paper, were the exposure(s) of interest measured prior to the outcome(s) being measured? | x |  |  |
| 1. Was the timeframe sufficient so that one could reasonably expect to see an association between exposure and outcome if it existed? | x |  |  |
| 1. For exposures that can vary in amount or level, did the study examine different levels of the exposure as related to the outcome (e.g., categories of exposure, or exposure measured as continuous variable)? | x |  |  |
| 1. Were the exposure measures (independent variables) clearly defined, valid, reliable, and implemented consistently across all study participants? | x |  |  |
| 1. Was the exposure(s) assessed more than once over time? |  | x |  |
| 1. Were the outcome measures (dependent variables) clearly defined, valid, reliable, and implemented consistently across all study participants? | x |  |  |
| 1. Were the outcome assessors blinded to the exposure status of participants? |  |  | x |
| 1. Was loss to follow-up after baseline 20% or less? |  |  | x |
| 1. Were key potential confounding variables measured and adjusted statistically for their impact on the relationship between exposure(s) and outcome(s)? | x |  |  |
| Author: Limpawattana *et al* 2017 | | | |
| Criteria | **Yes** | **No** | **Other (CD, NR, NA)*** |
| 1. Was the research question or objective in this paper clearly stated? | x |  |  |
| 1. Was the study population clearly specified and defined? | x |  |  |
| 1. Was the participation rate of eligible persons at least 50%? | x |  |  |
| 1. Were all the subjects selected or recruited from the same or similar populations (including the same time period)? Were inclusion and exclusion criteria for being in the study prespecified and applied uniformly to all participants? | x |  |  |
| 1. Was a sample size justification, power description, or variance and effect estimates provided? | x |  |  |
| 1. For the analyses in this paper, were the exposure(s) of interest measured prior to the outcome(s) being measured? |  |  | x |
| 1. Was the timeframe sufficient so that one could reasonably expect to see an association between exposure and outcome if it existed? | x |  |  |
| 1. For exposures that can vary in amount or level, did the study examine different levels of the exposure as related to the outcome (e.g., categories of exposure, or exposure measured as continuous variable)? | x |  |  |
| 1. Were the exposure measures (independent variables) clearly defined, valid, reliable, and implemented consistently across all study participants? | x |  |  |
| 1. Was the exposure(s) assessed more than once over time? |  | x |  |
| 1. Were the outcome measures (dependent variables) clearly defined, valid, reliable, and implemented consistently across all study participants? | x |  |  |
| 1. Were the outcome assessors blinded to the exposure status of participants? |  | x |  |
| 1. Was loss to follow-up after baseline 20% or less? |  |  | x |
| 1. Were key potential confounding variables measured and adjusted statistically for their impact on the relationship between exposure(s) and outcome(s)? | x |  |  |
| Author: Byun *et al*. 2017 | | | |
| Criteria | **Yes** | **No** | **Other (CD, NR, NA)*** |
| 1. Was the research question or objective in this paper clearly stated? | x |  |  |
| 1. Was the study population clearly specified and defined? | x |  |  |
| 1. Was the participation rate of eligible persons at least 50%? |  |  | x |
| 1. Were all the subjects selected or recruited from the same or similar populations (including the same time period)? Were inclusion and exclusion criteria for being in the study prespecified and applied uniformly to all participants? | x |  |  |
| 1. Was a sample size justification, power description, or variance and effect estimates provided? |  | x |  |
| 1. For the analyses in this paper, were the exposure(s) of interest measured prior to the outcome(s) being measured? | x |  |  |
| 1. Was the timeframe sufficient so that one could reasonably expect to see an association between exposure and outcome if it existed? | x |  |  |
| 1. For exposures that can vary in amount or level, did the study examine different levels of the exposure as related to the outcome (e.g., categories of exposure, or exposure measured as continuous variable)? | x |  |  |
| 1. Were the exposure measures (independent variables) clearly defined, valid, reliable, and implemented consistently across all study participants? | x |  |  |
| 1. Was the exposure(s) assessed more than once over time? |  |  | x |
| 1. Were the outcome measures (dependent variables) clearly defined, valid, reliable, and implemented consistently across all study participants? | x |  |  |
| 1. Were the outcome assessors blinded to the exposure status of participants? |  |  | x |
| 1. Was loss to follow-up after baseline 20% or less? |  |  | x |
| 1. Were key potential confounding variables measured and adjusted statistically for their impact on the relationship between exposure(s) and outcome(s)? | x |  |  |
| Author: Limpawattana *et al* 2017. | | | |
| Criteria | **Yes** | **No** | **Other (CD, NR, NA)*** |
| 1. Was the research question or objective in this paper clearly stated? | x |  |  |
| 1. Was the study population clearly specified and defined? | x |  |  |
| 1. Was the participation rate of eligible persons at least 50%? |  |  | x |
| 1. Were all the subjects selected or recruited from the same or similar populations (including the same time period)? Were inclusion and exclusion criteria for being in the study prespecified and applied uniformly to all participants? | x |  |  |
| 1. Was a sample size justification, power description, or variance and effect estimates provided? | x |  |  |
| 1. For the analyses in this paper, were the exposure(s) of interest measured prior to the outcome(s) being measured? |  |  | x |
| 1. Was the timeframe sufficient so that one could reasonably expect to see an association between exposure and outcome if it existed? | x |  |  |
| 1. For exposures that can vary in amount or level, did the study examine different levels of the exposure as related to the outcome (e.g., categories of exposure, or exposure measured as continuous variable)? | x |  |  |
| 1. Were the exposure measures (independent variables) clearly defined, valid, reliable, and implemented consistently across all study participants? | x |  |  |
| 1. Was the exposure(s) assessed more than once over time? |  |  | x |
| 1. Were the outcome measures (dependent variables) clearly defined, valid, reliable, and implemented consistently across all study participants? | x |  |  |
| 1. Were the outcome assessors blinded to the exposure status of participants? |  |  | x |
| 1. Was loss to follow-up after baseline 20% or less? |  |  | x |
| 1. Were key potential confounding variables measured and adjusted statistically for their impact on the relationship between exposure(s) and outcome(s)? | x |  |  |
| Author: Lee *et al*. 2017 | | | |
| Criteria | **Yes** | **No** | **Other (CD, NR, NA)*** |
| 1. Was the research question or objective in this paper clearly stated? | x |  |  |
| 1. Was the study population clearly specified and defined? | x |  |  |
| 1. Was the participation rate of eligible persons at least 50%? |  |  | x |
| 1. Were all the subjects selected or recruited from the same or similar populations (including the same time period)? Were inclusion and exclusion criteria for being in the study prespecified and applied uniformly to all participants? | x |  |  |
| 1. Was a sample size justification, power description, or variance and effect estimates provided? |  | x |  |
| 1. For the analyses in this paper, were the exposure(s) of interest measured prior to the outcome(s) being measured? |  |  | x |
| 1. Was the timeframe sufficient so that one could reasonably expect to see an association between exposure and outcome if it existed? | x |  |  |
| 1. For exposures that can vary in amount or level, did the study examine different levels of the exposure as related to the outcome (e.g., categories of exposure, or exposure measured as continuous variable)? | x |  |  |
| 1. Were the exposure measures (independent variables) clearly defined, valid, reliable, and implemented consistently across all study participants? | x |  |  |
| 1. Was the exposure(s) assessed more than once over time? |  |  | x |
| 1. Were the outcome measures (dependent variables) clearly defined, valid, reliable, and implemented consistently across all study participants? | x |  |  |
| 1. Were the outcome assessors blinded to the exposure status of participants? |  |  | x |
| 1. Was loss to follow-up after baseline 20% or less? |  |  | x |
| 1. Were key potential confounding variables measured and adjusted statistically for their impact on the relationship between exposure(s) and outcome(s)? | x |  |  |
| Author: Kneepers *et al*. 2017 | | | |
| Criteria | **Yes** | **No** | **Other (CD, NR, NA)*** |
| 1. Was the research question or objective in this paper clearly stated? | x |  |  |
| 1. Was the study population clearly specified and defined? | x |  |  |
| 1. Was the participation rate of eligible persons at least 50%? |  |  | x |
| 1. Were all the subjects selected or recruited from the same or similar populations (including the same time period)? Were inclusion and exclusion criteria for being in the study prespecified and applied uniformly to all participants? | x |  |  |
| 1. Was a sample size justification, power description, or variance and effect estimates provided? |  | x |  |
| 1. For the analyses in this paper, were the exposure(s) of interest measured prior to the outcome(s) being measured? |  |  | x |
| 1. Was the timeframe sufficient so that one could reasonably expect to see an association between exposure and outcome if it existed? |  |  | x |
| 1. For exposures that can vary in amount or level, did the study examine different levels of the exposure as related to the outcome (e.g., categories of exposure, or exposure measured as continuous variable)? | x |  |  |
| 1. Were the exposure measures (independent variables) clearly defined, valid, reliable, and implemented consistently across all study participants? | x |  |  |
| 1. Was the exposure(s) assessed more than once over time? |  |  | x |
| 1. Were the outcome measures (dependent variables) clearly defined, valid, reliable, and implemented consistently across all study participants? | x |  |  |
| 1. Were the outcome assessors blinded to the exposure status of participants? |  |  | x |
| 1. Was loss to follow-up after baseline 20% or less? |  |  | x |
| 1. Were key potential confounding variables measured and adjusted statistically for their impact on the relationship between exposure(s) and outcome(s)? | x |  |  |
| Author: Costa *et al*. 2017 | | | |
| Criteria | **Yes** | **No** | **Other (CD, NR, NA)*** |
| 1. Was the research question or objective in this paper clearly stated? | x |  |  |
| 1. Was the study population clearly specified and defined? | x |  |  |
| 1. Was the participation rate of eligible persons at least 50%? | x |  |  |
| 1. Were all the subjects selected or recruited from the same or similar populations (including the same time period)? Were inclusion and exclusion criteria for being in the study prespecified and applied uniformly to all participants? | x |  |  |
| 1. Was a sample size justification, power description, or variance and effect estimates provided? |  | x |  |
| 1. For the analyses in this paper, were the exposure(s) of interest measured prior to the outcome(s) being measured? |  |  | x |
| 1. Was the timeframe sufficient so that one could reasonably expect to see an association between exposure and outcome if it existed? | x |  |  |
| 1. For exposures that can vary in amount or level, did the study examine different levels of the exposure as related to the outcome (e.g., categories of exposure, or exposure measured as continuous variable)? | x |  |  |
| 1. Were the exposure measures (independent variables) clearly defined, valid, reliable, and implemented consistently across all study participants? | x |  |  |
| 1. Was the exposure(s) assessed more than once over time? |  |  | x |
| 1. Were the outcome measures (dependent variables) clearly defined, valid, reliable, and implemented consistently across all study participants? | x |  |  |
| 1. Were the outcome assessors blinded to the exposure status of participants? |  |  | x |
| 1. Was loss to follow-up after baseline 20% or less? |  |  | x |
| 1. Were key potential confounding variables measured and adjusted statistically for their impact on the relationship between exposure(s) and outcome(s)? | x |  |  |
| Author: Costa *et al*. 2018 | | | |
| Criteria | **Yes** | **No** | **Other (CD, NR, NA)*** |
| 1. Was the research question or objective in this paper clearly stated? | x |  |  |
| 1. Was the study population clearly specified and defined? | x |  |  |
| 1. Was the participation rate of eligible persons at least 50%? | x |  |  |
| 1. Were all the subjects selected or recruited from the same or similar populations (including the same time period)? Were inclusion and exclusion criteria for being in the study prespecified and applied uniformly to all participants? | x |  |  |
| 1. Was a sample size justification, power description, or variance and effect estimates provided? |  | x |  |
| 1. For the analyses in this paper, were the exposure(s) of interest measured prior to the outcome(s) being measured? |  |  | x |
| 1. Was the timeframe sufficient so that one could reasonably expect to see an association between exposure and outcome if it existed? | x |  |  |
| 1. For exposures that can vary in amount or level, did the study examine different levels of the exposure as related to the outcome (e.g., categories of exposure, or exposure measured as continuous variable)? | x |  |  |
| 1. Were the exposure measures (independent variables) clearly defined, valid, reliable, and implemented consistently across all study participants? | x |  |  |
| 1. Was the exposure(s) assessed more than once over time? |  | x |  |
| 1. Were the outcome measures (dependent variables) clearly defined, valid, reliable, and implemented consistently across all study participants? | x |  |  |
| 1. Were the outcome assessors blinded to the exposure status of participants? |  |  | x |
| 1. Was loss to follow-up after baseline 20% or less? |  |  | x |
| 1. Were key potential confounding variables measured and adjusted statistically for their impact on the relationship between exposure(s) and outcome(s)? | x |  |  |
| Author: De Blasio *et al*. 2018 | | | |
| Criteria | **Yes** | **No** | **Other (CD, NR, NA)*** |
| 1. Was the research question or objective in this paper clearly stated? | x |  |  |
| 1. Was the study population clearly specified and defined? | x |  |  |
| 1. Was the participation rate of eligible persons at least 50%? |  |  | x |
| 1. Were all the subjects selected or recruited from the same or similar populations (including the same time period)? Were inclusion and exclusion criteria for being in the study prespecified and applied uniformly to all participants? | x |  |  |
| 1. Was a sample size justification, power description, or variance and effect estimates provided? |  | x |  |
| 1. For the analyses in this paper, were the exposure(s) of interest measured prior to the outcome(s) being measured? |  |  | x |
| 1. Was the timeframe sufficient so that one could reasonably expect to see an association between exposure and outcome if it existed? |  |  | x |
| 1. For exposures that can vary in amount or level, did the study examine different levels of the exposure as related to the outcome (e.g., categories of exposure, or exposure measured as continuous variable)? | x |  |  |
| 1. Were the exposure measures (independent variables) clearly defined, valid, reliable, and implemented consistently across all study participants? | x |  |  |
| 1. Was the exposure(s) assessed more than once over time? |  |  | x |
| 1. Were the outcome measures (dependent variables) clearly defined, valid, reliable, and implemented consistently across all study participants? | x |  |  |
| 1. Were the outcome assessors blinded to the exposure status of participants? |  |  | x |
| 1. Was loss to follow-up after baseline 20% or less? |  |  | x |
| 1. Were key potential confounding variables measured and adjusted statistically for their impact on the relationship between exposure(s) and outcome(s)? | x |  |  |

| Table S3: Different cut-off points used to identify Sarcopenia. | | | |
| --- | --- | --- | --- |
| *Definition* | ***Lower MM*** | ***Lower MS*** | ***Lower PP*** |
| EWGSOP  2010 | ASMI: <7.23 kg·m−2 for men and <5.67 kg·m−2 for women. | HGS: <30 Kg for men and < 20 Kg for women. | GS ≤0.8 m/s |
| EWGSOP  2018 | ASM: ≤ 20 kg/m^2^ for men and ≤ 15 kg/ m^2^ for women. | HGS: <27 Kg for men and < 16 Kg for women. |  |
| AWGS | ASMI: ≤ 7.0 Kg/m^2^ for men and ≤ 5.4 Kg/m^2^ for women. | HGS: <26 Kg for men and < 18 Kg for women. |  |
| FNIH | ALM/BMI: <0.789 for men and for < 0.512 women. | HGS: <26 Kg for men and < 16 Kg for women. |  |
| AWGS: Asian Working Group of Sarcopenia; EWGSOP: European Working Group of Sarcopenia in Older People; FNIH: The Foundation for the National Institutes of Health; MM: muscle mass; MS: muscle strength; PP: physical performance. | | | |

**Figure S1: Prevalence of sarcopenia by gender.**


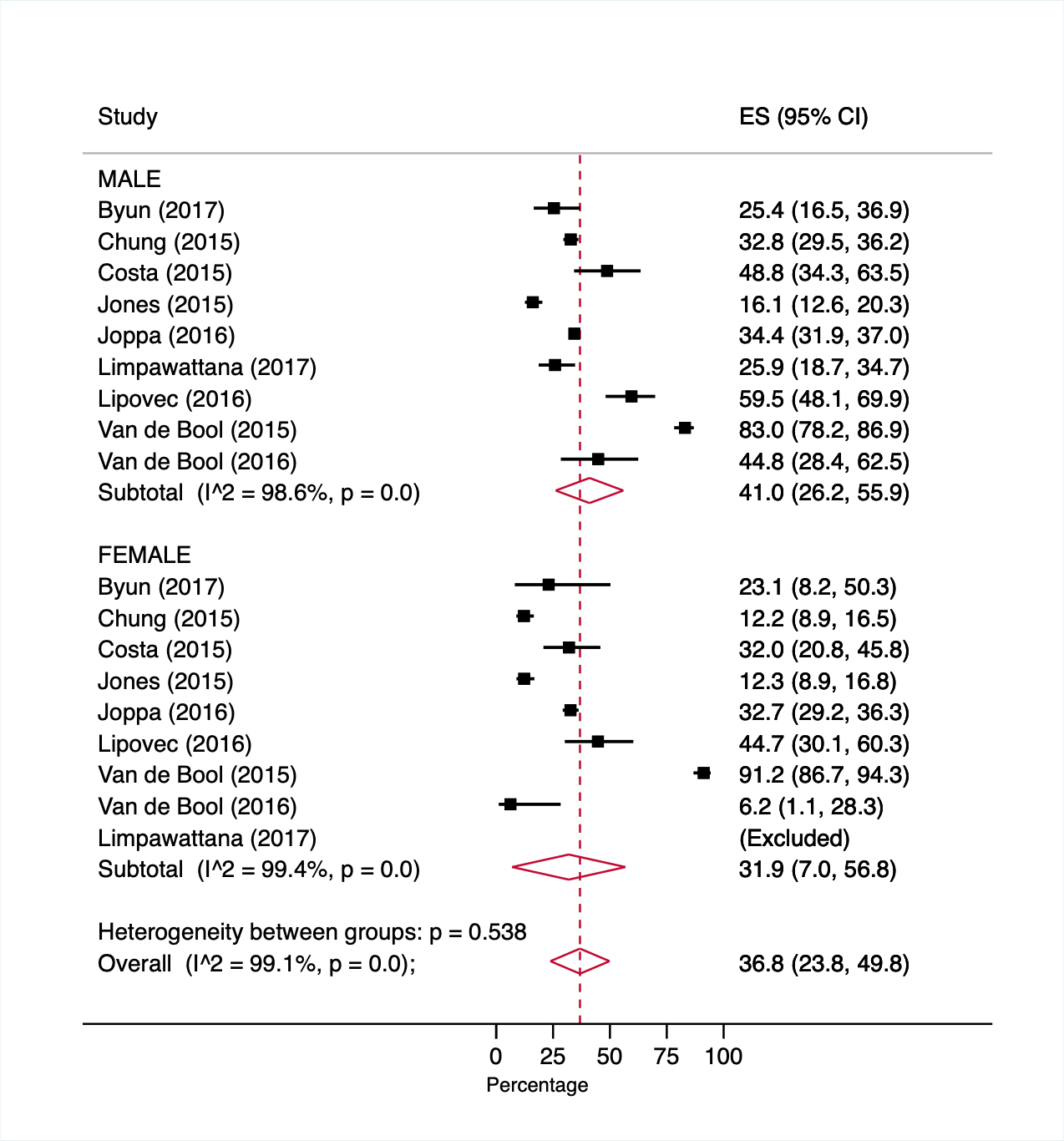


**ES = effect size (prevalence); I^2 = I^2^ heterogeneity statistic. Random effects model used for analysis, no significant difference between subgroups (p=0.5).**

**Figure S2. Meta-regression of effect of gender (percent male) on sarcopenia prevalence.**


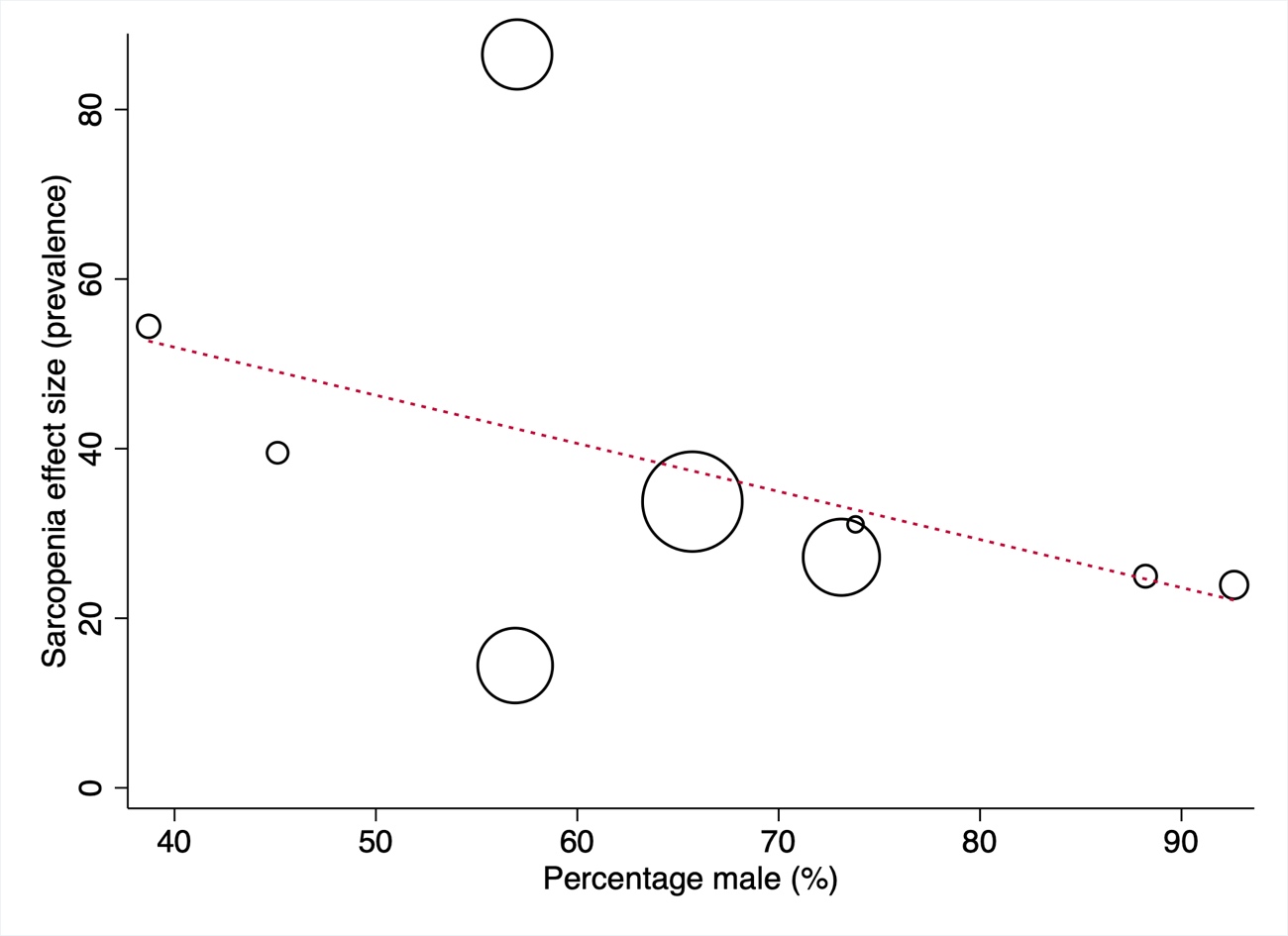


**Regression coefficient -0.006 (95%CI -0.015 to 0.004), p = 0.206. Circle diameters reflective of proportional study sample size.**

**Figure S3. Prevalence of sarcopenia, by COPD disease severity.**


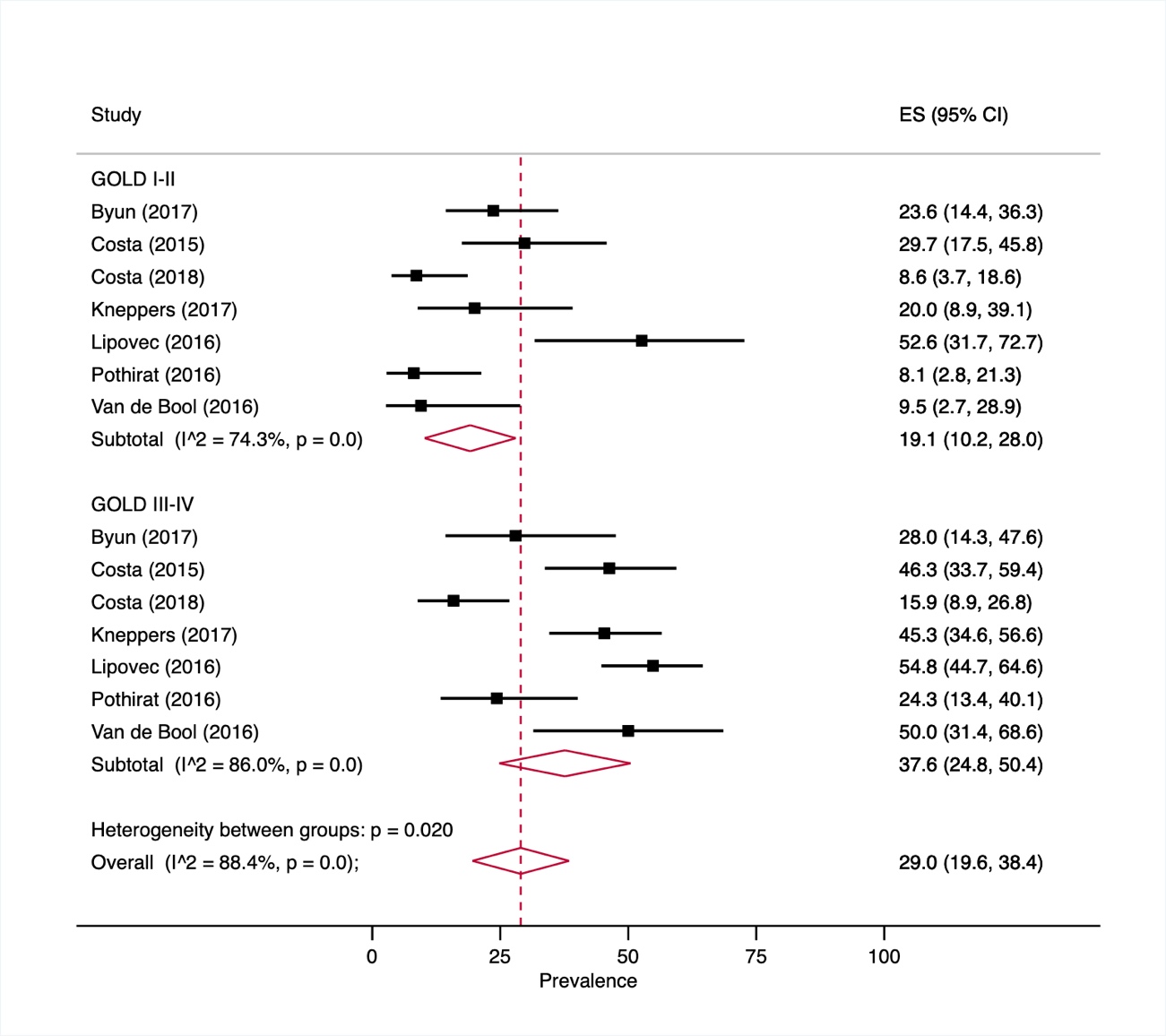


**ES = effect size (prevalence %); I^2 = I^2^ heterogeneity statistic. Random effects model used for analysis.**

**Figure S4. Meta-regression of effect of disease severity (GOLD stages III-IV) on sarcopenia prevalence.**


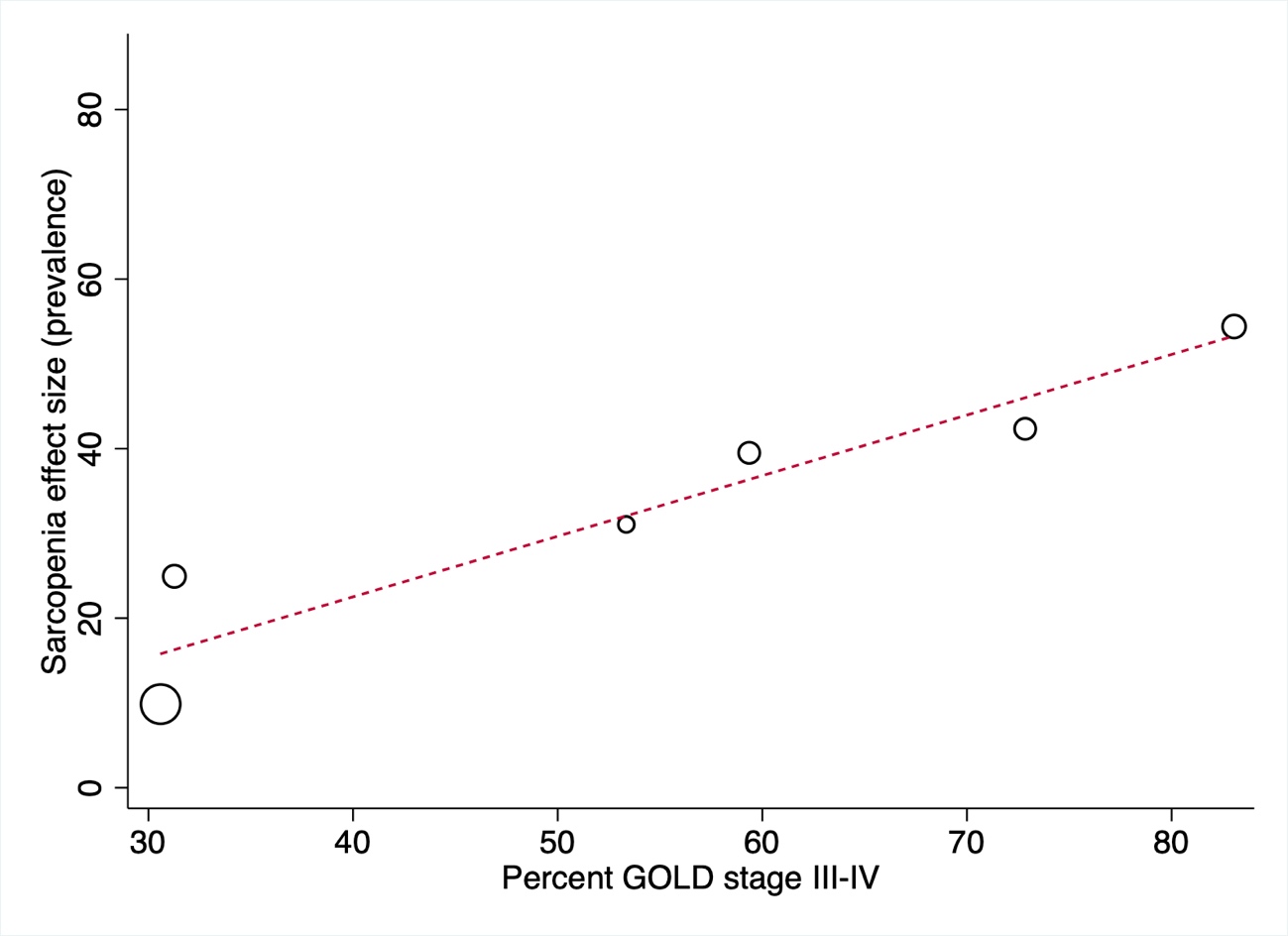


**Regression coefficient 0.715 (95%CI 0.342 to 1.088), p = 0.006; adjusted R^2^ = 90.1%.**

**Circle diameters reflective of proportional study sample size.**
